# Nitrogen-Doped Magnetic-Dielectric-Carbon Aerogel for High-Efficiency Electromagnetic Wave Absorption

**DOI:** 10.1007/s40820-023-01244-w

**Published:** 2023-11-17

**Authors:** Shijie Wang, Xue Zhang, Shuyan Hao, Jing Qiao, Zhou Wang, Lili Wu, Jiurong Liu, Fenglong Wang

**Affiliations:** 1 Key Laboratory for Liquid-Solid Structural Evolution and Processing of Materials Ministry of Education, Shandong University, Jinan, 250061 People’s Republic of China; 2 School of Mechanical Engineering, Shandong University, Jinan, 250061 People’s Republic of China; 3 Shenzhen Research Institute of Shandong University, Shenzhen, 518057 Guangdong People’s Republic of China

**Keywords:** Electromagnetic wave absorption, Wide bandwidth, Dielectric-magnetic synergy, Multifunction

## Abstract

Carbon-based aerogels derived from biomass chitosan are encountering a flourishing moment in electromagnetic protection on account of lightweight, controllable fabrication and versatility. Nevertheless, developing a facile construction method of component design with carbon-based aerogels for high-efficiency electromagnetic wave absorption (EWA) materials with a broad effective absorption bandwidth (EAB) and strong absorption yet hits some snags. Herein, the nitrogen-doped magnetic-dielectric-carbon aerogel was obtained via ice template method followed by carbonization treatment, homogeneous and abundant nickel (Ni) and manganese oxide (MnO) particles in situ grew on the carbon aerogels. Thanks to the optimization of impedance matching of dielectric/magnetic components to carbon aerogels, the nitrogen-doped magnetic-dielectric-carbon aerogel (Ni/MnO-CA) suggests a praiseworthy EWA performance, with an ultra-wide EAB of 7.36 GHz and a minimum reflection loss (RL_min_) of − 64.09 dB, while achieving a specific reflection loss of − 253.32 dB mm^−1^. Furthermore, the aerogel reveals excellent radar stealth, infrared stealth, and thermal management capabilities. Hence, the high-performance, easy fabricated and multifunctional nickel/manganese oxide/carbon aerogels have broad application aspects for electromagnetic protection, electronic devices and aerospace.

**Supplementary Information:**

The online version contains supplementary material available at 10.1007/s40820-023-01244-w.

## Highlights

- An ingenious design achieved magnetic-dielectric-carbon coupling.
- Nickel and manganese oxide particles were in situ reduced and grew on the carbon aerogels.
- The aerogels demonstrated radar stealth, infrared stealth and thermal management capability.

**Supplementary Information:**

The online version contains supplementary material available at 10.1007/s40820-023-01244-w.

## Graphical abstract

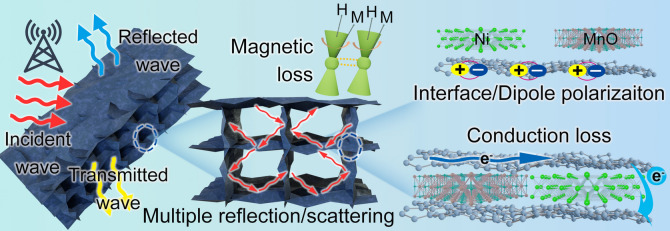

## Introduction

Gigahertz band wireless communication technology has brought great convenience to human life, but the impact of electromagnetic pollution on human health and environment cannot be ignored [1, 2]. Electronic equipment used in aerospace, radar and computer system is closely related to daily life of human beings, so electromagnetic wave absorption (EWA) materials with high performance, facile fabrication and environmentally friendly need to be developed to solve the matters of electromagnetic pollution [3, 4].

EWA materials, by absorbing the incident electromagnetic waves and converting electromagnetic energy into Joule heat and releasing it into the surrounding environment, so as to achieve the purpose of attenuating electromagnetic waves [5, 6]. As an EWA material with excellent performance, minimum reflection loss (RL_min_) and effective absorption bandwidth (EAB) are the basic criteria for evaluating its characteristic [7]. With the evolution of miniature and lightweight electronic equipment, especially in the fields of aviation and aerospace, low density and thin thickness have become two critical requirements [8, 9]. Not only that, considering the complex practical application environment of the material, the absorber is usually coated on the surface of the equipment, so the thermal insulation ability of the material needs to be further improved in order to reduce the temperature changes on the performance of the equipment [10, 11]. Obviously, conventional powder materials cannot cope with the above requirements. With the deepening of research, the high specific surface area and rich porosity of foams, sponges and aerogels themselves have given the materials the ability to be lightweight and thermal insulating, which makes them potential candidates among absorbing materials.

In recent years, biomass-derived carbon aerogels have developed vigorously in the field of electromagnetic compatibility and protection due to wide range of sources, diversity of morphology and structure, and facile component combination [12, 13]. Due to their high porosity, large specific surface and abundant functional groups, carbon aerogels provide a variety of interfaces and polarization sites for the effective attenuation of electromagnetic waves. However, the impedance mismatch caused by excessive electrical conductivity and single dielectric loss make carbon aerogels still unsatisfactory [14, 15]. Using the adjustable morphological structure, the magnetic/dielectric components are combined with carbon aerogels, which may give the aerogels materials suitable impedance matching and synergetic magnetic loss, so as to realize the preparation of broadband EWA materials [16, 17]. Nickel, as a typical soft magnetic material, exhibits a higher saturation magnetization strength compared to most ferrite materials, which is beneficial in enhancing magnetic loss. Ni is conducive to raising the Snoek’s limit at high frequency and enhancing the magnetic loss ability of materials due to high permeability and magnetization strength [5, 18]. As a cost-effective dielectric material, manganese oxide (MnO) has excellent stability and unique physical and chemical properties. Its low dielectric constant can be used to adjust the impedance matching of carbon aerogels, inducing more incident electromagnetic waves to enter the aerogel interior. MnO with low conductivity can adjust the overall conductivity of composite materials while suppressing eddy current loss of magnetic materials [19]. Chitin is a biological resource whose content is second only to cellulose in nature, and it has rich nitrogen content [20, 21]. The introduction of nitrogen atoms can cause various defects in the carbon skeleton, such as dislocation and lamellar bending. The difference in electronegativity between nitrogen and carbon atoms changes the distribution of electron clouds and leads to the formation of electric dipoles in the electromagnetic field, inducing dipole polarization and contributing to the dissipation of electromagnetic waves [14, 22]. Chitosan, deacetylation of chitin, can be dissolved into acidic solution to form stable suspension and then prepared into aerogel by freeze drying. Therefore, chitosan has received extensive attention and as a biomass resource for the preparation of three-dimensional porous carbon aerogels [23, 24]. Chitosan-derived carbon aerogels have received widespread attention in the electromagnetic field due to their wide availability, environmentally-friendly nature, and controllable preparation. However, the electromagnetic wave absorption properties of aerogels previously studied are not outstanding, including narrow band width and weak absorption capacity [14, 25]. Limited by the acidic treatment conditions of chitosan, the typical combination method of impregnation is used, but the method can lead to weak binding and uneven distribution between components and carbon aerogels. At present, the implementation of the above attributes still faces huge challenges.

In this work, we have fabricated a nitrogen-doped multi-component carbon aerogel (Ni/MnO-CA) by facile freeze-drying and carbonization, and realized in situ composite of magnetic/dielectric components with carbon aerogel. The Ni and MnO modulated the impedance matching of the carbon aerogel, and the synergistic effect of dielectric and magnetic loss allowed Ni/MnO-CA to obtain an impressive EWA performance with RL_min_ of − 64.09 dB and EAB of 7.36 GHz. The three-dimensional cell structure and the synergy of multiple components made aerogels versatile, such as considerable radar stealth, infrared stealth, and thermal management capabilities. Therefore, compared with the same type of absorbers, the magnetic-dielectric-carbon aerogel of this work has significant advantages in the field of electromagnetic protection and electronic device applications.

## Experimental Section

### Materials

Chitosan ((C_6_H_11_NO_4_)n, < 200 mPa s) and glacial acetic acid were bought from Shanghai Aladdin Biochemical Technology Co., Ltd. Nickel (II) chloride hexahydrate (NiCl_2_·6H_2_O) and manganese (II) chloride tetrahydrate (MnCl_2_·4H_2_O) were purchased from Sinopharm Chemical Reagent Co., Ltd. All the chemicals were analytically pure without any other treatment.

### Fabrication of Magnetic-Dielectric-Carbon Aerogels

The fabrication process of aerogels included directed freezing, freeze drying and carbonization. In the first step, 0.75 g of chitosan powder was dissolved in 30 mL of deionized water and magnetically stirred for 20 min. Subsequently, 1.05 mmol NiCl_2_·6H_2_O and 1.05 mmol MnCl_2_·4H_2_O were added to the above solution and magnetically stirred for 10 min. After that, 0.51 mL of glacial acetic acid was quickly dripped into the solution to form a suspension. Then, the suspension was slowly poured into a homemade Teflon mold with copper base (4 × 4 × 4 cm^3^) and placed together in liquid nitrogen (− 196 ℃) for directed freezing. After 24 h of freeze-drying, the xerogel was carbonized under nitrogen atmosphere (700 ℃) to obtain magnetic-dielectric-carbon aerogel (Ni/MnO-CA). For comparison, we also prepared the Ni-CA and MnO-CA via the similar procedure, without introducing Mn or Ni sources in the precursor, where the total content of the precursor metal salts was maintained as 2.1 mmol. In addition, in order to explore the influence of magnetic/dielectric components on electromagnetic parameters, the total content of precursor metal salts (the content of NiCl_2_·6H_2_O and MnCl_2_·4H_2_O was 1:1) was increased to 3.15 and 4.2 mmol, and the final aerogels were named after Ni/MnO-CA-1.5 and Ni/MnO-CA-2.0, respectively.

### Characterizations

The morphology and microstructure were recorded by field emission scanning electron microscopy (FE-SEM; Hitachi, Model SU-70) and high solution electron microscope (HRTEM, JEM 2100F). The X-ray photoelectron spectroscopy (XPS) were tested by photoelectron spectrometer (Thermo ESCALAB 250Xi). The X-ray diffraction (XRD) spectra were recorded by powder X-ray diffractometer (DMAX-2500PC, Rigaku). Fourier transform infrared spectra (FT-IR) were recorded on Nicolet iS 5 FT-IR spectroscopic analyzer. Thermogravimetric analysis (TGA, HCT-1) was used to assess the content of carbon. Nitrogen adsorption–desorption isotherms were recorded by specific surface area and porosity analyzer (ASAP2460). The magnetic loops at room temperature were recorded by a vibration sample magnetometer (VSM, LakeShore 7404). The zeta potential was determined by using a potentiometric analyzer (Malvern, UK). The Raman spectra were through by a Raman spectrometer (LabRam HR Evolution). Thermal conductivity at room temperature was obtained by LFA 467. Infrared emissivity values in 3–5 and 8–14 μm wavelengths were recorded by China-Wanyi-IR2 type dual-band emissivity measuring instrument. Thermal images of the aerogels were obtained from a FLIR E86 infrared thermal camera and FLIR Tools + software was used to deal with the data. The electromagnetic parameters were measured by a vector network analyzer (VNA; Agilent PNA N5244A). Here, a coaxial ring method was applied, and an annulus sample with 10 wt% aerogel fragment and 90 wt% paraffin mixing was made by a tailored metallic mold.

## Results and Discussion

### Composition and Structure

**Fig. 1:**
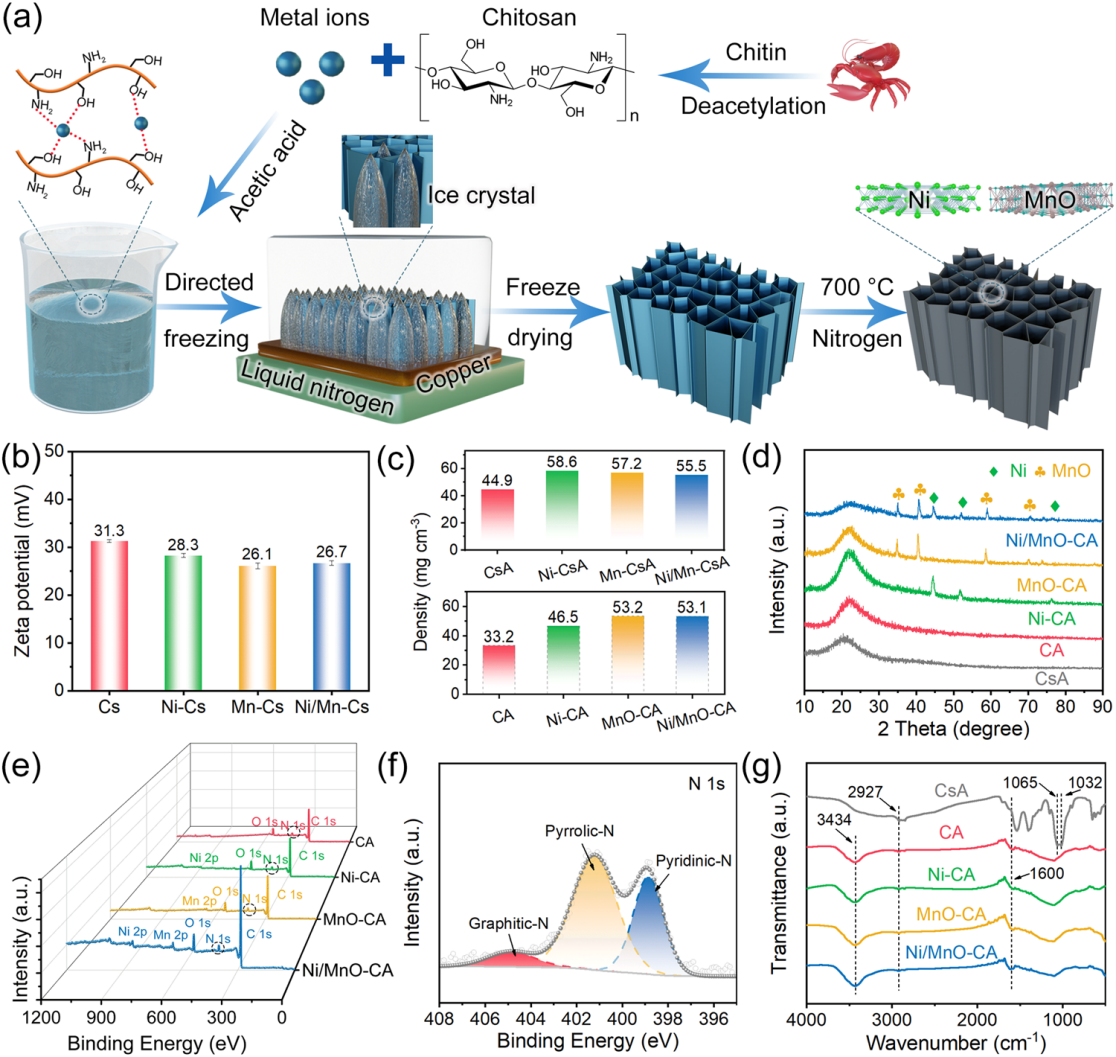
**a** Schematic illustration of fabricating Ni/MnO-CA. **b** Zeta potential of chitosan suspension before and after Ni/Mn ion addition. **c** Density of aerogels before and after carbonization. **d** XRD patterns of CsA, CA, Ni-CA, MnO-CA, and Ni/MnO-CA. XPS spectra of **e** CA, Ni-CA, MnO-CA, and Ni/MnO-CA and **f** N 1*s* region of Ni/MnO-CA. **g** FT-IR

The fabrication process of nitrogen-doped magnetic-dielectric-carbon aerogel (Ni/MnO-CA) is depicted in the figure (Fig. 1a), including freeze-drying and subsequent carbonization process. Initially, NiCl_2_·6H_2_O, MnCl_2_·4H_2_O and chitosan powder were dissolved in an aqueous solution of glacial acetic acid at room temperature to form a homogeneous and stable suspension (Ni/Mn-Cs, Fig. S1, Tyndall effect), which demonstrated the good stability and dispersion. Due to the protonation of a large number of free amino groups in the chitosan molecular chain, long-chain polymer chitosan could be dissolved in glacial acetic acid solution to form a homogeneous suspension [26]. The introduction of Ni and Mn ions reduced the zeta potential of the suspension slightly, but the suspension still maintained good stability (Figs. 1b and S2). In particular, the transition metal ions would react with the amino and hydroxyl groups on the chitosan molecular chain, which could promote the cross-linking of molecular and maintain the stability of the suspension [27]. Subsequently, the suspension was freeze-dried and the xerogel was obtained to test the FT-IR (Fig. S3). For pure chitosan xerogels (CsA), the peaks at 1533 and 1396 cm^−1^ corresponded to the stretching vibration peaks of amide –NH_2_ and C–N bonds [15, 20]. Analysis of the curves of xerogels with Ni and Mn ions revealed that the above two peaks underwent blue shift, which proved that transition metal ions induced complexation of chitosan chains. During directed freezing, a large number of ice crystals grew vertically because of the temperature gradient, and the suspension located at the front of the ice crystals solidified to form a continuous cell wall [28, 29]. After freeze-drying, ice crystals sublimated and escaped to form xerogel (Ni/Mn-CsA, Fig. S4). Finally, under the protective atmosphere of nitrogen, chitosan was pyrolyzed into amorphous carbon and reducing gas [30], and nickel and manganese ions were reduced to metal nickel and manganese oxide uniformly distributed on the carbon skeleton via carbothermal reaction. Herein, anisotropic N-doped magnetic-dielectric-carbon aerogel (Ni/MnO-CA) was successfully prepared. The density of aerogels before and after nitrogen treatment is shown in Fig. 1c, and XRD further analyzed the component transformation in Fig. 1d. After CsA was carbonized, the wide peak of the carbon aerogel (CA) at 22.6° corresponded to the (002) crystal plane of graphite [31, 32]. In addition, the characteristic peaks of 44.5°, 51.8°, and 76.4° corresponded to (111), (200), and (220) planes of Ni (No. 87–0712), the peaks at 35.0°, 40.7°, 58.9°, and 70.4° were assigned to (111), (200), (200), (220), and (311) planes of MnO (No. 75–0626), respectively, which confirmed the successful in situ reduction in metal nickel and manganese oxide [33, 34].

The surface chemistry of magnetic-dielectric-carbon aerogels was meticulously analyzed by XPS. Since chitosan was rich in amino functional groups, nitrogen doping was obtained after carbonization, and the spectrum of XPS further confirmed the existence of nitrogen (Fig. 1e). In the N 1*s* orbit (Fig. 1f), doped nitrogen atoms existed in the form of pyridinic N (398.9 eV), pyrrolic N (401.2 eV) and graphitic N (404.8 eV) [22], which favored the conductivity loss of carbon materials because nitrogen doping could effectively reduce the intrinsic resistance and promote electron transport. In particular, pyridinic N provided unpaired electrons to form π-conjugated rings with *sp*^*2*^ hybrid carbon atoms, which was conducive to enhancing electron transport and graphitic N replaced carbon atoms in the graphite layer and introduced delocalized π electrons, which reduced the intrinsic resistance of carbon materials. Moreover, based on the C 1*s* orbit (Fig. S5a), the deconvolution peaks of 284.6, 285.6, and 288.8 eV were consistent with C–C, C–N, and C = O bonds, respectively, where oxygen-containing groups were derived from remnants of chitosan pyrolysis [35, 36]. After pyrolysis, most hydrophilic groups in chitosan were reduced, such as –COC, –COH, and –CH_2_OH in the range of 1200–1000 cm^−1^. And due to the acid treatment moved from 3434 to 2927 cm^−1^, the –OH vibration peak was also narrowed and the group was reduced. However, the peak of the C=O bond of the amide at 1600 cm^–1^ was more pronounced (Fig. 1g). The oxygen groups would induce dipole polarizations to promote electromagnetic wave attenuation. In addition, the XPS spectra of O 1*s*, Ni 2*p*, and Mn 2*p* are depicted in Fig. S5b–d, which once again revealed the presence of metal nickel and manganese oxide.

**Fig. 2:**
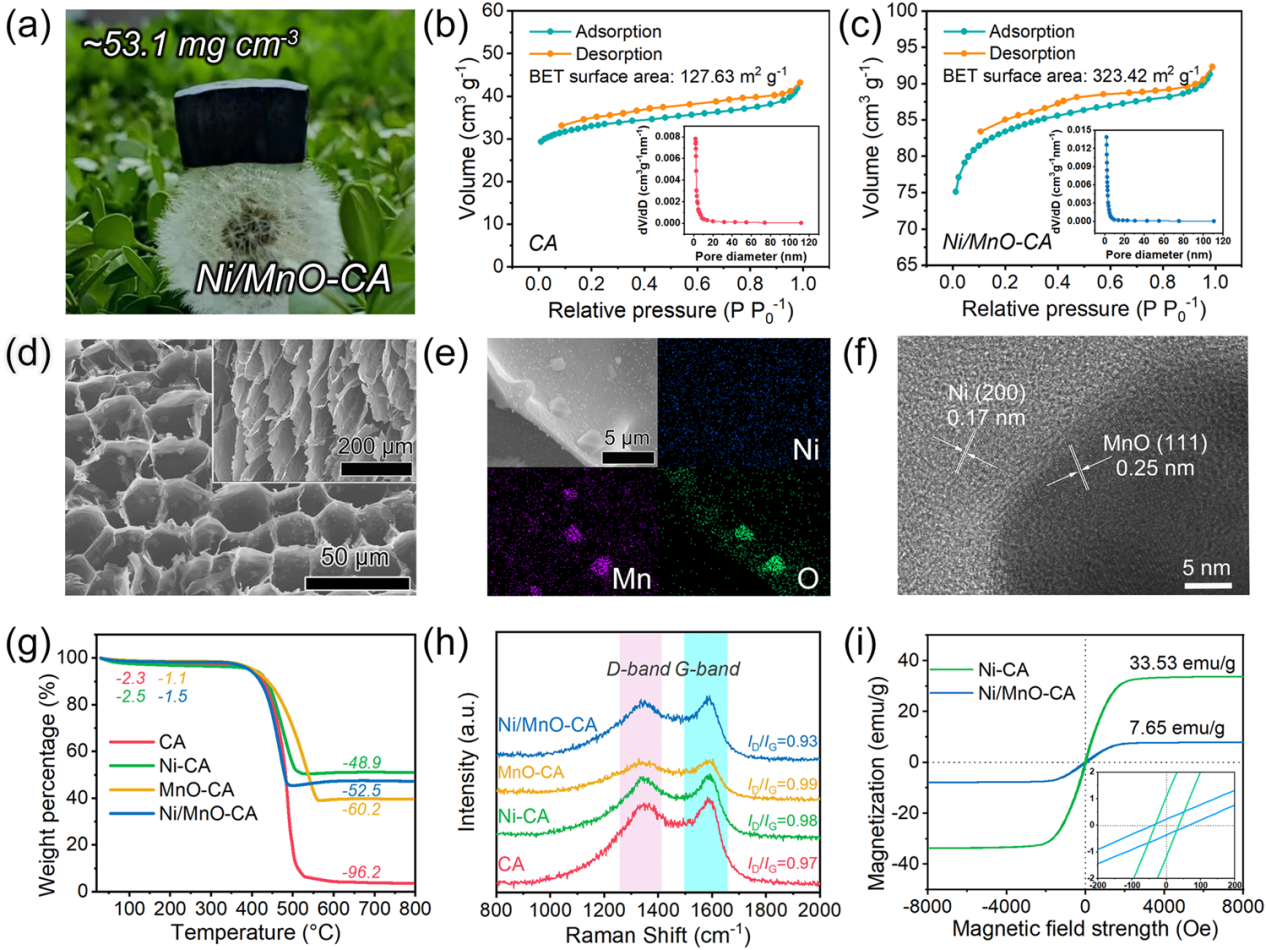
**a** Digital photograph of Ni/MnO-CA standing on a dandelion. BET surface area and pore size distribution of **b** CA and **c** Ni/MnO-CA. **d** Field emission scanning electron microscope (FE-SEM) of Ni/MnO-CA, the inset was parallel to the direction of ice crystal. Ni/MnO-CA: **e** EDS mappings and **f** High-resolution TEM image. **g** TGA curves, **h** Raman spectra and **i** Magnetic hysteresis loops

The N-doped magnetic-dielectric-carbon aerogel prepared in situ had a lower density (~ 53.1 mg cm^−3^), and lightweight properties allow it to stand on a dandelion (Fig. 2a). Compared with carbon aerogel, in situ grown particles increased the specific surface area of the aerogel (Figs. 2b, c and S6a, b), and the nitrogen absorption and desorption curves confirmed that Ni/MnO-CA had a high BET specific surface area (323.42 m^2^ g^−1^) and mesoporous presence [37]. In addition, the macroscopic pore size and microstructure of Ni/MnO-CA were confirmed by scanning electron microscopy (SEM). All aerogels exhibited three-dimensional porous structures (Figs. 2d and S7), which provided favorable channels for multiple reflection and scattering of electromagnetic waves [38, 39]. Further, the lamellar structures were arranged parallel to the direction of ice crystal growth, with pores of about 25 μm perpendicular to the direction of ice crystal growth, and Ni and MnO particles were evenly distributed on them. Based on transmission electron microscopy (TEM) analysis, the interplanar spacing of 0.17 and 0.25 nm belonged to Ni (200) and MnO (111) lattice plane, respectively. The close bonding of Ni and MnO particles with carbon could improve the interface quality and facilitate polarization. Analyzing the TGA curves (Fig. 2g), the decline in the first stage (less than 150 °C) belonged to the evaporation of the absorbed water, and the second stage came from the decomposition of the carbon support [15, 40]. Ignoring the adsorbed water, we determined the amount of carbon in the aerogel composites with the help of formulas (Eqs. S1–S3). The carbon content in Ni-CA, MnO-CA, and Ni/MnO-CA was 59.85, 62.98, and 57.51 wt%, respectively, indicating that the carbon content in aerogel composites was similar. For carbon-based absorbing materials, the degree of graphitization of carbon affected the conductivity of the material, which in turn affected the EWA performance of the material [41, 42]. The *I*_D_/*I*_G_ ratio in the Raman curve was used to assess the degree of graphitization of carbon [43], in which the D band (an active *A*_1g_ mode of crystallite boundaries) at around 1340 cm^–1^ was thought to be the existence of disorders and defects, and the G band (from an active *E*_2g_ mode of infinite crystals) at 1590 cm^–1^ corresponded to the perfect graphite lattice regions [44, 45]. The four carbon aerogel composites had a comparable degree of graphitization (Fig. 2h), revealing that the introduction of Ni or MnO components did not destroy the degree of graphitization of the carbon matrix [46, 47]. The introduction of magnetic Ni notably affected the magnetism of aerogel composites, as shown in Fig. 2i. Ni-CA possessed a high saturation magnetization of 33.53 emu g^−1^ and the value of Ni/MnO-CA was 7.65 emu g^−1^. In addition, the coercivity of the two was 33.2 and 42.1 Oe, respectively, which were higher than that of bulk nickel (~ 0.7 Oe) [10]. The large coercivity would lead to increased magnetic anisotropy, which caused the natural resonance to move towards higher frequency and improved the magnetic loss performance in GHz frequency range.

### Electromagnetic Wave Absorption Properties

**Fig. 3:**
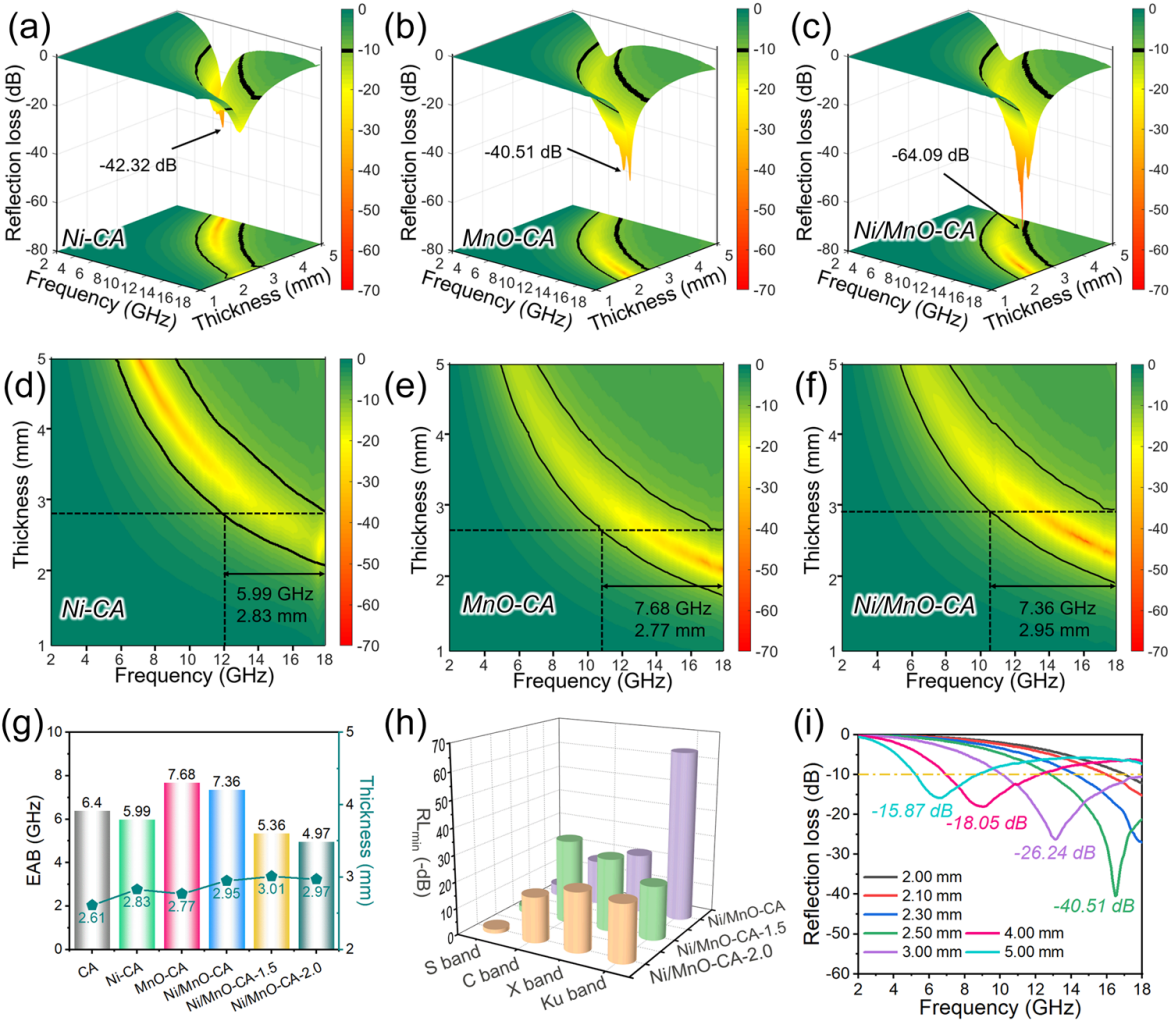
3D RL plots of **a** Ni-CA, **b** MnO-CA and **c** Ni/MnO-CA. 2D contours of RL values versus frequency and thickness of **d** Ni-CA, **e** MnO-CA and **f** Ni/MnO-CA. **g** EAB and corresponding thickness for the aerogels. **h** RL_min_ values of the aerogels at different frequency bands. **i** 1D curves of RL values versus frequency at different thicknesses for Ni/MnO-CA

According to the transmission line theory, the electromagnetic wave absorption performance of magnetic-dielectric-carbon aerogels was expressed by the reflection loss [22, 48] (Eqs. S4–S5). The absorption capacity of CA for electromagnetic wave was not commendable, featuring an RL_min_ of − 18.31 dB and an EAB of 6.4 GHz (Fig. S8). Typically, a reflection loss value of less than − 10 dB meant that 90% of the incident electromagnetic waves were attenuated and dissipated in the form of heat [11, 25]. However, the loss capability of CA was insufficient for the absorbers with excellent performance. Obviously, the in situ introduction of magnetic Ni and transition metal oxide MnO improved the loss capability of aerogel composites for electromagnetic waves (Fig. 3a–f). In detail, Ni-CA had an RL_min_ value of − 42.32 dB while maintaining an EAB of 5.99 GHz, and MnO-CA had a slightly reduced RL_min_ value of − 40.51 dB, but the EAB reached 7.68 GHz. Interestingly, the coexistence of Ni and MnO made Ni/MnO-CA obtain the outstanding EWA performance, in which RL_min_ was − 64.09 dB and a wide EAB of 7.36 GHz. Further, we increased the ratio of Ni and MnO in aerogel composites, and found that their ability to lose electromagnetic waves decreased, and the frequency range with reflection losses below − 10 dB was narrowed (Fig. S9). More intuitively, the EAB and corresponding matching thickness of the above aerogel composites were compared (Fig. 3g). Despite the slight advantages and disadvantages of the loss capability of electromagnetic waves, aerogel materials exhibited a wide EAB and a thin matching thickness, which was good for absorbers. Subsequently, for aerogel composites with different Ni/MnO contents, we compared the minimum reflection loss in different frequency bands, and it could be found that with the increase in component content, the strongest absorption of electromagnetic waves by aerogels generally moved towards the low-frequency region (Fig. 3h). In addition, Ni/MnO-CA absorbed more than 90% of electromagnetic waves at different thicknesses, demonstrating its excellent absorption performance (Fig. 3i).

### Electromagnetic Parameters

**Fig. 4:**
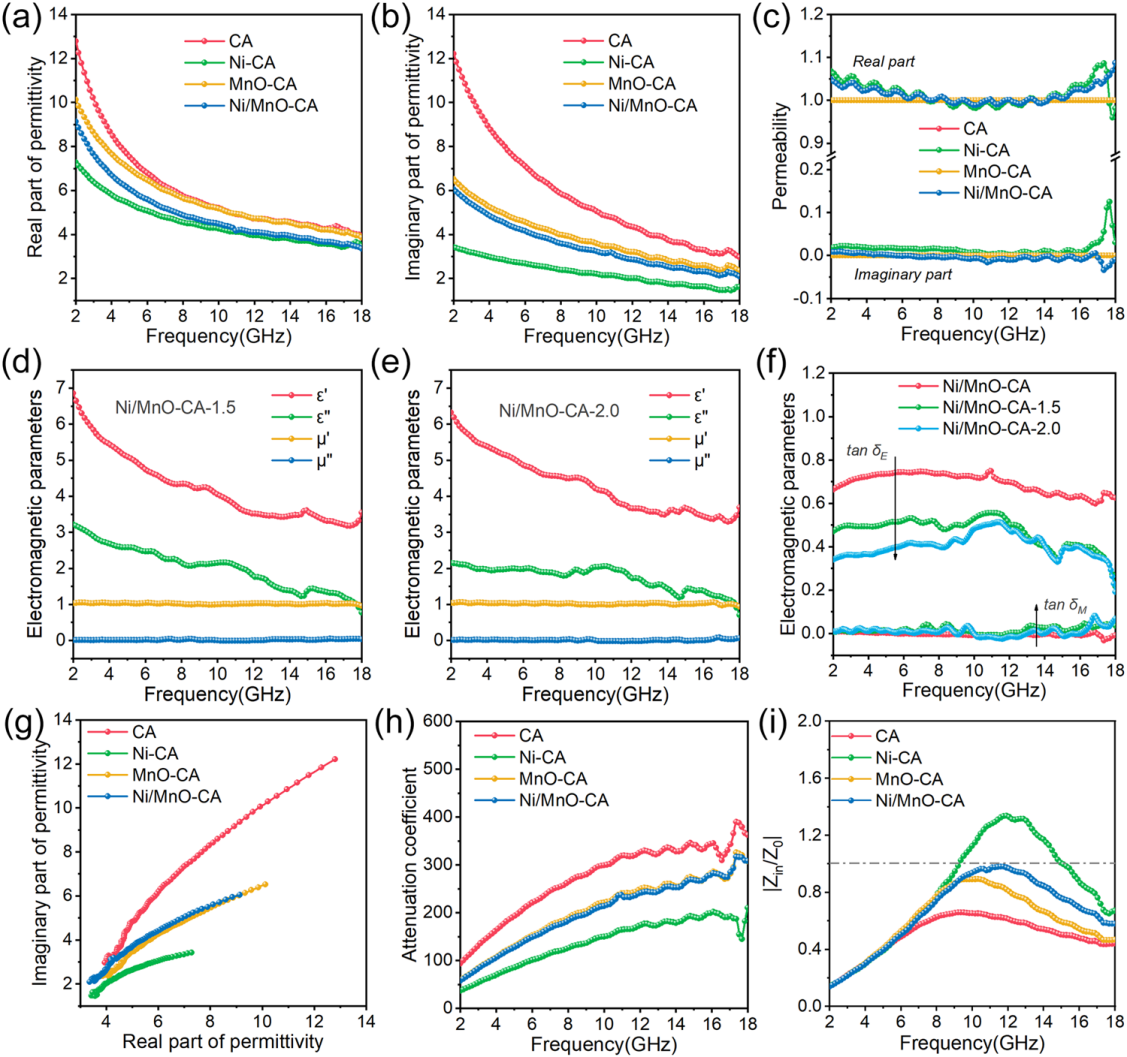
**a** Real part of permittivity. **b** Imaginary part of permittivity and **c** permeability. Electromagnetic parameters of **d** Ni/MnO-CA-1.5 and **e** Ni/MnO-CA-2.0. **(f)** The loss tangent. **g** The Cole–Cole plot. **h** Attenuation coefficient and **i** impedance matching values

Aiming at deeply exploring the mechanism of the above aerogels, it was crucial to measure the electromagnetic parameters, which include complex permittivity $\left( {\varepsilon_{r} = \varepsilon^{\prime } {-} \, j\varepsilon^{\prime \prime } } \right)$ and complex permeability $\left( {\mu_{{\text{r}}} = \mu^{\prime } {-} \, j\mu^{\prime \prime } } \right)$. In general, the real parts of permittivity (*εʹ*) and permeability (*μʹ*) represented the ability to store electrical and magnetic energy, and correspondingly, the imaginary parts (*εʹʹ*, *μʹʹ*) indicated the ability to dissipate energy [49]. The permittivity of aerogels showed a decreasing trend with increasing frequency (Fig. 4a), which was consistent with the frequency dispersion behaviors of carbon materials [50, 51]. The *εʹ* and *εʹʹ* value of CA decreased from 12.8 to 4.0 and from 12.2 to 3.0 (Fig. 4b), respectively, indicating the dominant contribution of conductive losses. According to the free electron theory (*εʹʹ* = *σ*/(2*πε*_0_*f*)), the imaginary part of permittivity was related to the conductivity of materials [52]. For CA obtained by carbonized chitosan under N_2_ atmosphere, its imaginary part of permittivity was too high, which was due to excessive conductivity. Too high a permittivity was not conducive to impedance matching, resulting in strong reflection of electromagnetic waves on the surface of the absorbers, so the EWA performance of CA was insufficient. In more detail, the *εʹʹ* of Ni-CA and MnO-CA was from 3.4 to 1.6 and 6.5 to 2.4. The in-situ introduction of the components Ni and MnO inhibited the electrical conductivity of the composite aerogel thereby reducing the imaginary part of permittivity. Magnetic nickel particles gave aerogels the ability to store and dissipate magnetic energy, which was manifested by the change of permeability with frequency (Fig. 4c). The curve in the imaginary part had obvious peaks at high frequency, indicating the presence of magnetic resonance loss, which could be understood as exchange resonances. In addition, we analyzed the contribution of eddy current loss to magnetic loss by plotting the *C*_0_ curve (Fig. S10a). Typically, if eddy current loss was present in magnetic loss, the *C*_0_ was constant [53]. In the test frequency range, the *C*_0_ values decreased with increasing frequency, showing that the eddy current loss in magnetic materials was suppressed and magnetic resonance dominated magnetic loss [54]. By plotting the tangent curves of dielectric loss and magnetic loss (Fig. S10b–c), the dielectric and magnetic loss capabilities were evaluated. By comparison, the dielectric loss contributed greatly to the attenuation of electromagnetic waves. When the content of Ni/MnO increased, the dielectric loss capacity of aerogels decreased, and the magnetic loss increased (Fig. 4d–f), which ultimately led to a decrease in EWA performance. Therefore, dielectric loss in the loss mechanism had a greater impact on performance [55, 56].

When considering the attenuation characteristics of materials, dielectric loss included conductivity loss and dielectric relaxation loss [57, 58]. In the Cole–Cole plot (Fig. 4g, Eqs. S6–S8), the long and tall tail indicated the conductivity loss of the four aerogels, and the CA was significantly higher than the other three. A few semicircles at the curve indicated that the material had dipole polarization and interfacial polarization [4]. Specifically, the C = O functional groups and C–N bonds derived from chitosan contributed to the dipole polarization. The interface between particles and carbon constituted the interfacial polarization in the aerogels. In order to further evaluate the comprehensive attenuation ability of aerogels to electromagnetic waves, we discussed the attenuation coefficient (α, Eq. S9). The introduction of Ni or MnO reduced the attenuation ability of carbon aerogels to electromagnetic waves, in which Ni-CA had the weakest ability, and both MnO-CA and Ni/MnO-CA had the similar capabilities (Fig. 4h). However, Ni/MnO-CA possessed the best EWA performance, so we further analyzed the impedance matching coefficient (*Z,* Fig. 4i). When *Z* value was closer to 1, the excellent impedance matching could be achieved, so the reflection at the interface of absorber was minimized. Although the loss capability of aerogels to electromagnetic waves was a key factor affecting performance, it could not be ignored that electromagnetic waves could effectively enter the absorber and then be dissipated when the absorber was adapted to the impedance of space. CA has the strongest attenuation capability, but its performance was not the best due to its impedance mismatch, resulting in too many electromagnetic waves being reflected on the surface of absorbers. The in-situ recombination of Ni or MnO with carbon aerogels improved the impedance matching of materials, as shown in the figure (Fig. S11). It could be noted that the EWA performance of Ni/MnO-CA was more prominent, because the introduction of magnetic nickel further improved the impedance matching between the aerogels and space, and the magnetic loss brought by it achieved the best EWA performance.

**Fig. 5:**
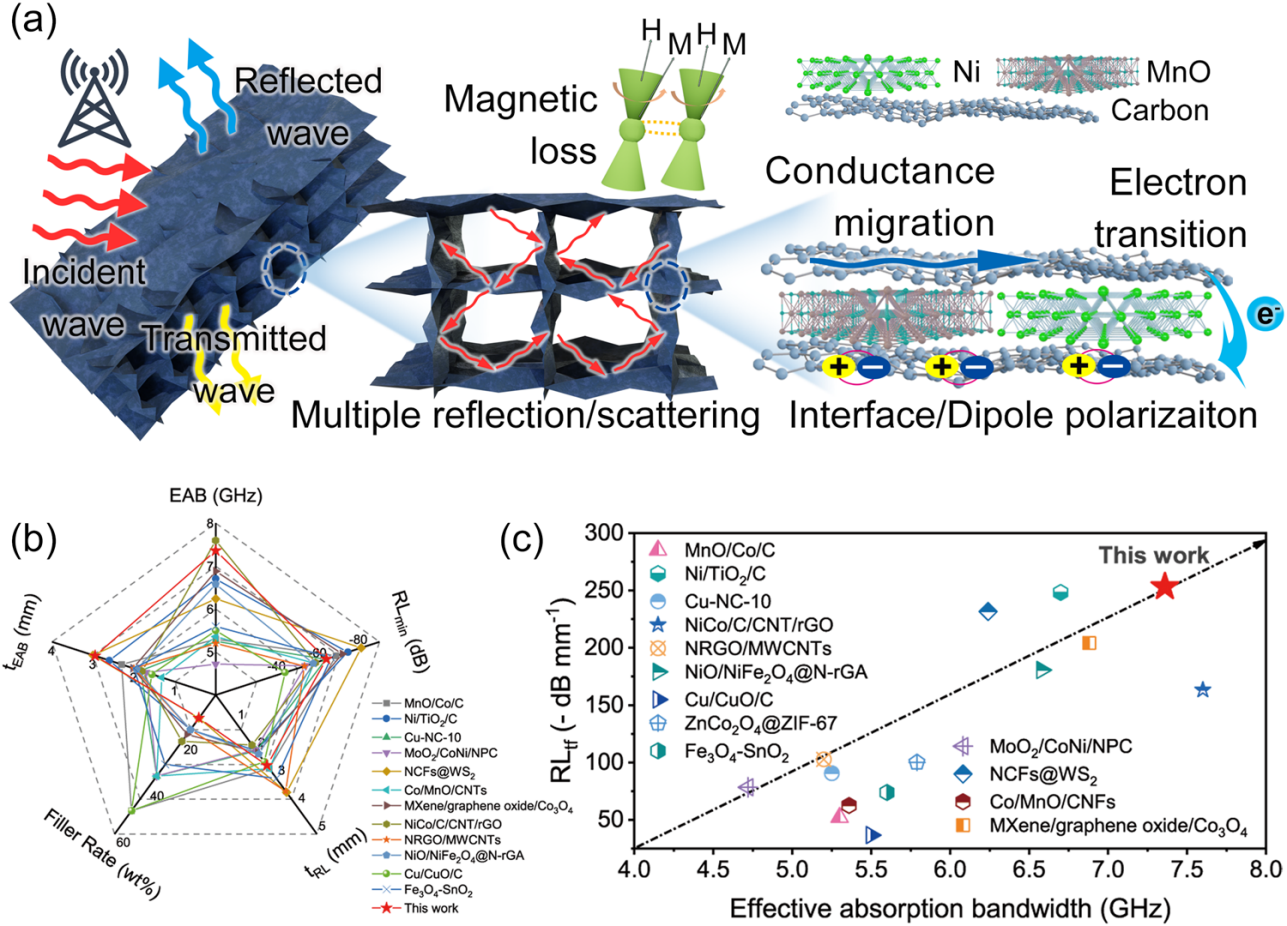
**a** Schematic diagram of EWA mechanisms for Ni/MnO-CA. **b** Radar chart of the properties. **c** Comparation of RL_tf_ values and EAB with previous works

Based on the above discussion, the possible interaction mechanism of magnetic-dielectric-carbon aerogels with electromagnetic waves was shown (Fig. 5a). Firstly, the porous structure of aerogels provided a transmission channel, which was conducive to multiple reflections and scattering of electromagnetic waves in the internal space structure of the aerogels to achieve the attenuation of electromagnetic waves [59]. Secondly, the combination of Ni/MnO with carbon aerogels regulated the impedance matching of aerogels and free space, so that the electromagnetic waves could enter into aerogels more effectively and interact with them. Based on electron migration in graphite crystallite and electron transitions between crystallite, conductive loss in aerogel contributed to the attenuation of electromagnetic waves. Finally, Ni/MnO-CA achieved excellent EWA performance due to the synergistic effect of dielectric and magnetic loss, conduction loss by electron migration of carriers in the conductive carbon layer, dipole polarization and interface polarization of different components, and resonance in magnetic loss [60].

In comparison with the EWA materials in the previous literature (Table S1), Ni/MnO-CA demonstrated the wide EAB of 7.36 GHz, strong absorption of − 64.09 dB and low filler rate (10 wt%), as shown in radar chart (Fig. 5b). Moreover, RL_tf_ was used to explain the RL_min_ *vs* thickness and filler rate, further highlight the lightness, thinness, width, and strength of excellent EWA materials [16, 18]. While maintaining a wide EAB, the RL_tf_ of this work reached − 253.32 dB mm^−1^, which had a significant advantage over the same type of absorbers (Fig. 5c).

### Electromagnetic Wave Absorption by CST Simulation

**Fig. 6:**
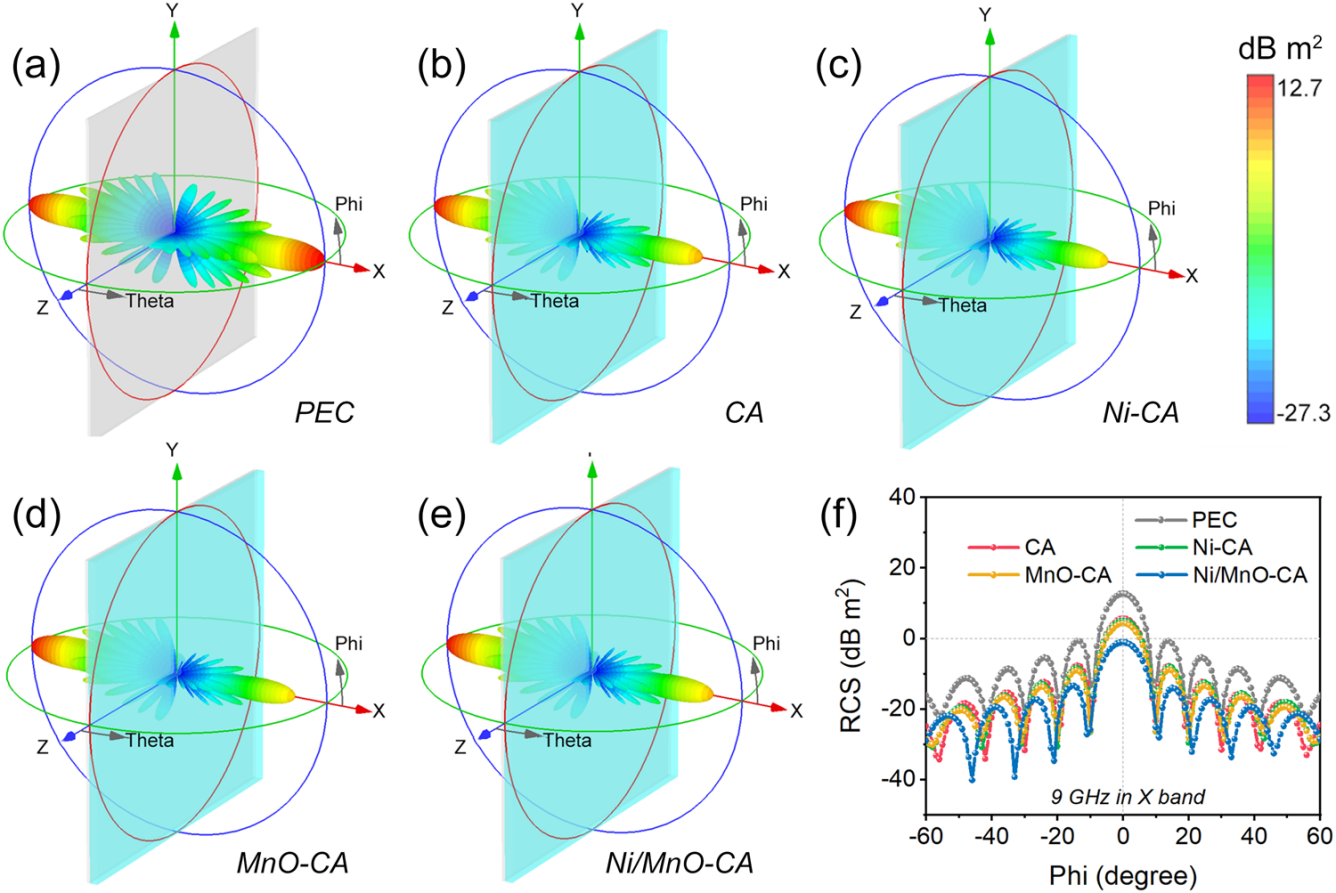
3D far-field response of RCS simulations at 9 GHz. **a** PEC, **b** CA, **c** Ni-CA, **d** MnO-CA and **e** Ni/MnO-CA. **f** 1D plot of RCS simulated values

To further verify the stealth performance of aerogels in practical applications, we simulated the far-field response in radar cross section (RCS) by *CST microwave studio*. At the 9 GHz monitoring frequency in X band, the 3D far-field simulation plots of perfect electric conductor and four aerogels are shown in Fig. 6a–e, and we also gave the results at the monitoring frequency in other bands (Figs. S13–S14). RCS values of materials stood for the physical quantity of the echo intensity under radar detection, therefore the smaller the values, the better the stealth performance [61]. The maximum RCS values for the PEC model, CA, Ni-CA, MnO-CA, and Ni/MnO-CA composites were 12.7, 5.62, 5.11, 4.31, and − 1.06 dB m^2^, respectively. These simulated results were consistent with the EWA performance of each aerogel material. Compared with PEC and three other aerogels, Ni/MnO-CA demonstrated its fantastic radar stealth performance in practical applications (Fig. 6f).

### Infrared Stealth Performance and Thermal Management Capability

**Fig. 7:**
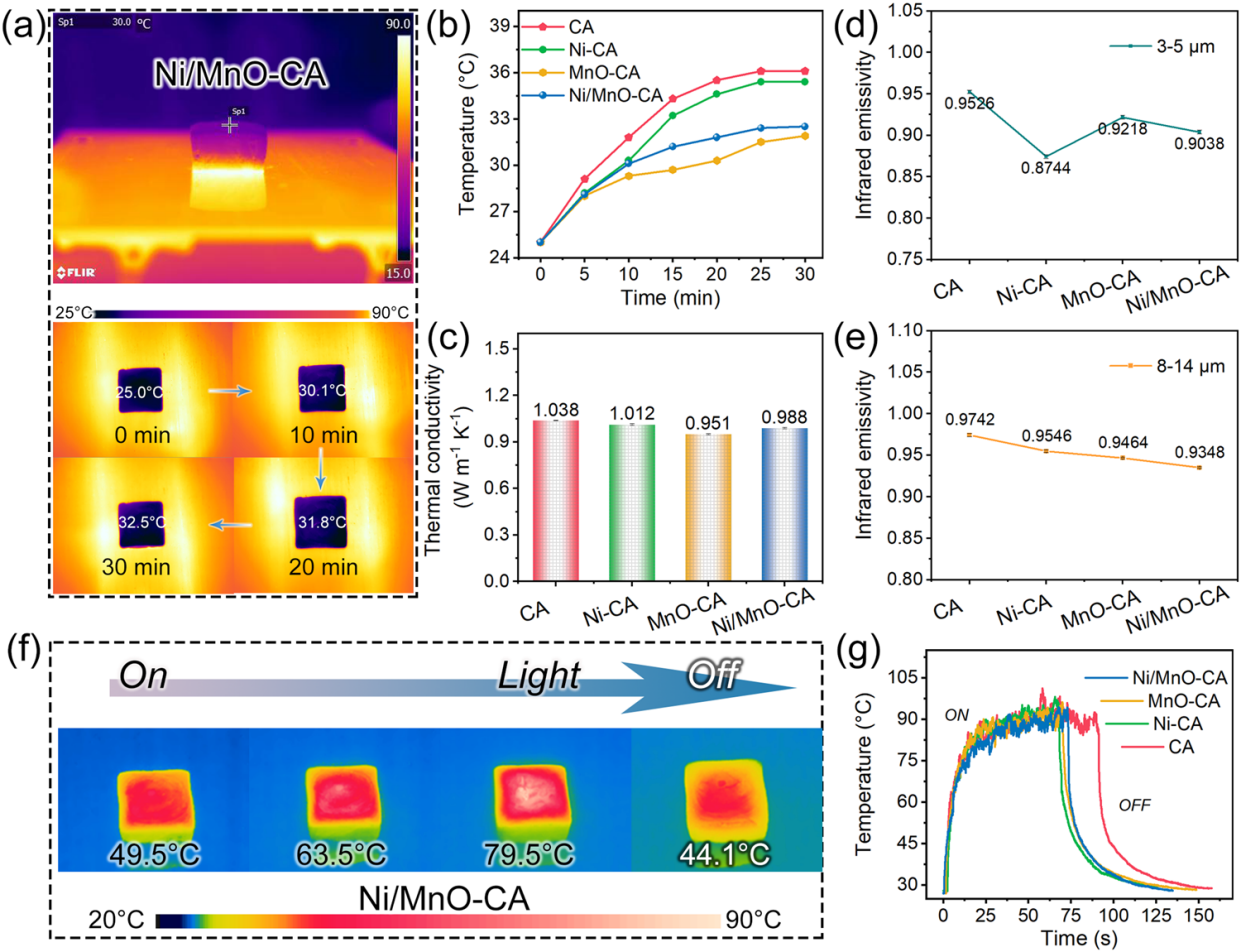
**a** Infrared thermal images of Ni/MnO-CA on a constant temperature heating plate of 90 ℃. **b** Temperature–time curves of aerogels surface. **c** Thermal conductivity of CA, Ni-CA, MnO-CA and Ni/MnO-CA. Infrared emissivity of **d** 3–5 μm and **e** 8–14 μm. **f** Infrared images of solar-thermal of Ni/MnO-CA. **g** Photothermal curves of aerogels, and the simulated sunlight used was 180 mW cm^−2^

In order to cope with complex and changeable application environments, the versatility of EWA materials was worth exploring. Therein, infrared stealth performance was vital in practical applications, and excellent stealth performance could reduce the risk of being detected by infrared detectors [62]. Hence, we tested the thermal insulation performance of aerogels and infrared emissivity of different wavelength bands. First, the temperature change of the aerogel surface was recorded with an infrared camera by placing the aerogel (2.45 × 2.45 × 0.95 cm^3^) on a 90 °C heating plate (Figs. 7a and S15–S17). After about 30 min, all aerogels showed excellent thermal insulation properties, where the surface temperature of CA was only 36.1 °C, while the thermal insulation ability of the other three aerogels was further improved (Fig. 7b). The thermal conductivity of the above aerogels was shown in the figure (Fig. 7c), the outstanding thermal insulation ability was due to the air inside the aerogel and the pores of the skeleton hindering heat conduction. Second, we tested the infrared emissivity of aerogels in the two atmospheric window regions of 3–5 and 8–14 μm in infrared detection, as shown in the figure (Fig. 7d–e). The overall infrared emissivity of aerogels at 3–5 μm was lower than that of 8–14 μm, and the lower emissivity confirmed the brilliant infrared stealth performance of aerogels. In addition, Ni/MnO-CA showed good photothermal conversion ability (Fig. 7f), which could reach 85 ℃ with 35 s under simulated sunlight of 180 mW cm − ^2^ (Fig. 7g). The photothermal conversion ability made aerogel materials excellent thermal management ability, which depicted that it had great potential in the field of electronic devices.

## Conclusion

In summary, the nitrogen-doped magnetic-dielectric-carbon aerogel (Ni/MnO-CA) was facilely fabricated by ice template method and carbonization treatment. The reasonable component design and pore structure endowed the aerogel efficient EWA performance, and the radar, infrared stealth and thermal management capabilities made it suitable for more complex and changeable environments. The in situ introduction of nickel and manganese oxide components adjusted the impedance matching of the carbon aerogel, so Ni/MnO-CA obtained an RL_min_ of − 64.09 dB and a specific absorption intensity of − 253.32 dB mm^−1^, while maintaining an ultra-wide EAB of 7.36 GHz at a matching thickness of 2.95 mm. Further, RCS far-field simulation revealed its considerable radar stealth performance, while its outstanding thermal insulation ability and low infrared emissivity confirmed its excellent infrared stealth performance. In addition, the rapid photothermal conversion further demonstrated good thermal management capabilities. High-performance, easily fabricated and multifunctional Ni/MnO-CA possessed broad application prospects for electromagnetic compatibility protection, electronics and aerospace.

## Supplementary Information

Below is the link to the electronic supplementary material.

Supplementary file1 (PDF 1691 kb)

## References

[1] X. Zhang, J. Qiao, Y. Jiang, F. Wang, X. Tian et al., Carbon-based mof derivatives: emerging efficient electromagnetic wave absorption agents. Nano-Micro Lett. **13**(1), 135 (2021). 10.1007/s40820-021-00658-8 10.1007/s40820-021-00658-8 PMC8180543 34138364

[2] H. Lv, Z. Yang, H. Pan, R. Wu, Electromagnetic absorption materials: current progress and new frontiers. Prog. Mater. Sci. **127**, 100946 (2022). 10.1016/j.pmatsci.2022.100946

[3] W. Gu, S.J.H. Ong, Y. Shen, W. Guo, Y. Fang et al., A lightweight, elastic, and thermally insulating stealth foam with high infrared-radar compatibility. Adv. Sci. **9**(35), e2204165 (2022). 10.1002/advs.202204165 10.1002/advs.202204165 PMC9762302 36285685

[4] Y. Li, X. Liu, X. Nie, W. Yang, Y. Wang et al., Multifunctional organic–inorganic hybrid aerogel for self-cleaning, heat-insulating, and highly efficient microwave absorbing material. Adv. Funct. Mater. **29**(10), 1807624 (2019). 10.1002/adfm.201807624

[5] X. Meng, J. Qiao, S. Zheng, H. Tian, B. Li et al., Ternary nickel/molybdenum dioxide/carbon composited nanofibers for broadband and strong electromagnetic wave absorption. Chem. Eng. J. **457**, 141241 (2023). 10.1016/j.cej.2022.141241

[6] S. Gao, G.S. Wang, L. Guo, S.H. Yu, Tunable and ultraefficient microwave absorption properties of trace n-doped two-dimensional carbon-based nanocomposites loaded with multi-rare earth oxides. Small **16**(19), e1906668 (2020). 10.1002/smll.201906668 32297713 10.1002/smll.201906668

[7] F. Pan, X. Wu, D. Batalu, W. Lu, H. Guan, Assembling of low-dimensional aggregates with interlaminar electromagnetic synergy network for high-efficient microwave absorption. Adv. Powder Mater. **2**(2), 100100 (2023). 10.1016/j.apmate.2022.100100

[8] H. Jiang, L. Cai, F. Pan, Y. Shi, J. Cheng et al., Ordered heterostructured aerogel with broadband electromagnetic wave absorption based on mesoscopic magnetic superposition enhancement. Adv. Sci. (2023). 10.1002/advs.202301599 10.1002/advs.202301599 PMC10375159 37150852

[9] L. Lyu, F. Wang, X. Zhang, J. Qiao, C. Liu et al., CuNi alloy/carbon foam nanohybrids as high-performance electromagnetic wave absorbers. Carbon **172**, 488–496 (2021). 10.1016/j.carbon.2020.10.021

[10] Z. Wang, L. Yang, Y. Zhou, C. Xu, M. Yan et al., NiFe LDH/MXene derivatives interconnected with carbon fabric for flexible electromagnetic wave absorption. ACS Appl. Mater. Interfaces **13**(14), 16713–16721 (2021). 10.1021/acsami.1c05007 33818065 10.1021/acsami.1c05007

[11] J. Qiao, X. Zhang, C. Liu, Z. Zeng, Y. Yang et al., Facile synthesis of mns nanoparticle embedded porous carbon nanocomposite fibers for broadband electromagnetic wave absorption. Carbon **191**, 525–534 (2022). 10.1016/j.carbon.2022.02.024

[12] Z. Guo, P. Ren, F. Yang, T. Wu, L. Zhang et al., MOF-derived Co/C and MXene co-decorated cellulose-derived hybrid carbon aerogel with a multi-interface architecture toward absorption-dominated ultra-efficient electromagnetic interference shielding. ACS Appl. Mater. Interfaces **15**(5), 7308–7318 (2023). 10.1021/acsami.2c22447 36693013 10.1021/acsami.2c22447

[13] C. Cui, L. Geng, S. Jiang, W. Bai, L. Dai et al., Construction of hierarchical carbon fiber Aerogel@Hollow Co_9_S_8_ polyhedron for high-performance electromagnetic wave absorption at low-frequency. Chem. Eng. J. **466**, 143122 (2023). 10.1016/j.cej.2023.143122

[14] Y. Tian, D. Estevez, H. Wei, M. Peng, L. Zhou et al., Chitosan-derived carbon aerogels with multiscale features for efficient microwave absorption. Chem. Eng. J. **421**, 129781 (2021). 10.1016/j.cej.2021.129781

[15] X. Chen, M. Zhou, Y. Zhao, W. Gu, Y. Wu et al., Morphology control of eco-friendly chitosan-derived carbon aerogels for efficient microwave absorption at thin thickness and thermal stealth. Green Chem. **24**(13), 5280–5290 (2022). 10.1039/d2gc01604d

[16] L. Liang, Q. Li, X. Yan, Y. Feng, Y. Wang et al., Multifunctional magnetic Ti_3_C_2_T_x_ MXene/graphene aerogel with superior electromagnetic wave absorption performance. ACS Nano **15**(4), 6622–6632 (2021). 10.1021/acsnano.0c09982 33780231 10.1021/acsnano.0c09982

[17] F. Gan, Q. Rao, J. Deng, L. Cheng, Y. Zhong et al., Controllable architecture of ZnO/FeNi composites derived from trimetallic ZnFeNi layered double hydroxides for high-performance electromagnetic wave absorbers. Small (2023). 10.1002/smll.202300257 10.1002/smll.202300257 36967536

[18] J. Qiao, X. Zhang, C. Liu, L. Lyu, Z. Wang et al., Facile fabrication of Ni embedded TiO_2_/C core-shell ternary nanofibers with multicomponent functional synergy for efficient electromagnetic wave absorption. Compos. Part B Eng. **200**, 108343 (2020). 10.1016/j.compositesb.2020.108343

[19] D. Xu, Y. Yang, L. Lyu, A. Ouyang, W. Liu et al., One-dimensional MnO@N-doped carbon nanotubes as robust dielectric loss electromagnetic wave absorbers. Chem. Eng. J. **410**, 128295 (2021). 10.1016/j.cej.2020.128295

[20] X. Chen, Z. Wang, M. Zhou, Y. Zhao, S. Tang et al., Multilevel structure carbon aerogels with 99999% electromagnetic wave absorptivity at 18 mm and efficient thermal stealth. Chem. Eng. J. **452**, 139110 (2023). 10.1016/j.cej.2022.139110

[21] H. Ababneh, B.H. Hameed, Chitosan-derived hydrothermally carbonized materials and its applications: a review of recent literature. Int. J. Biol. Macromol. **186**, 314–327 (2021). 10.1016/j.ijbiomac.2021.06.161 34197858 10.1016/j.ijbiomac.2021.06.161

[22] M. Qin, L. Zhang, H. Wu, Dielectric loss mechanism in electromagnetic wave absorbing materials. Adv. Sci. **9**(10), e2105553 (2022). 10.1002/advs.202105553 10.1002/advs.202105553 PMC8981909 35128836

[23] J. Hu, C. Zhao, Y. Si, C. Hong, Y. Xing et al., Preparation of ultrafine microporous nitrogen self-doped chitosan (CS) carbon aerogel based on a Zn-Zn system for high-performance supercapacitors. Appl. Surf. Sci. **637**, 157910 (2023). 10.1016/j.apsusc.2023.157910

[24] Y. Gao, Z. Lei, L. Pan, Q. Wu, X. Zhuang et al., Lightweight chitosan-derived carbon/rGO aerogels loaded with hollow Co_1-x_Ni_x_O nanocubes for superior electromagnetic wave absorption and heat insulation. Chem. Eng. J. **457**, 141325 (2023). 10.1016/j.cej.2023.141325

[25] L. Wang, M. Liu, G. Wang, B. Dai, F. Yu et al., An ultralight nitrogen-doped carbon aerogel anchored by Ni-NiO nanoparticles for enhanced microwave adsorption performance. J. Alloys Compd. **776**, 43–51 (2019). 10.1016/j.jallcom.2018.10.214

[26] A. Varamesh, B.D. Abraham, H. Wang, P. Berton, H. Zhao et al., Multifunctional fully biobased aerogels for water remediation: applications for dye and heavy metal adsorption and oil/water separation. J. Hazard. Mater. **457**, 131824 (2023). 10.1016/j.jhazmat.2023.131824 37327610 10.1016/j.jhazmat.2023.131824

[27] Z. Sun, F. Lv, L. Cao, L. Liu, Y. Zhang et al., Multistimuli-responsive, moldable supramolecular hydrogels cross-linked by ultrafast complexation of metal ions and biopolymers. Angew. Chem. Int. Ed. **54**(27), 7944–7948 (2015). 10.1002/anie.201502228 10.1002/anie.201502228 26012538

[28] S. Liu, S. Wang, M. Sang, J. Zhou, J. Zhang et al., Nacre-mimetic hierarchical architecture in polyborosiloxane composites for synergistically enhanced impact resistance and ultra-efficient electromagnetic interference shielding. ACS Nano **16**(11), 19067–19086 (2022). 10.1021/acsnano.2c08104 36302097 10.1021/acsnano.2c08104

[29] H. Joukhdar, A. Seifert, T. Jungst, J. Groll, M.S. Lord et al., Ice templating soft matter: fundamental principles and fabrication approaches to tailor pore structure and morphology and their biomedical applications. Adv. Mater. **33**(34), e2100091 (2021). 10.1002/adma.202100091 34236118 10.1002/adma.202100091

[30] M. Wang, J. Zhang, X. Yi, X. Zhao, B. Liu et al., Nitrogen-doped hierarchical porous carbon derived from ZIF-8 supported on carbon aerogels with advanced performance for supercapacitor. Appl. Surf. Sci. **507**, 145166 (2020). 10.1016/j.apsusc.2019.145166

[31] B. Dai, F. Dong, H. Wang, Y. Qu, J. Ding et al., Fabrication of CuS/Fe_3_O_4_@polypyrrole flower-like composites for excellent electromagnetic wave absorption. J. Colloid Interface Sci. **634**, 481–494 (2023). 10.1016/j.jcis.2022.12.029 36542977 10.1016/j.jcis.2022.12.029

[32] H. Han, X. Xu, H. Kan, Y. Tang, C. Liu et al., Synergistic photodynamic/photothermal bacterial inactivation over heterogeneous quaternized chitosan/silver/cobalt phosphide nanocomposites. J. Colloid Interface Sci. **616**, 304–315 (2022). 10.1016/j.jcis.2022.02.068 35219196 10.1016/j.jcis.2022.02.068

[33] G. Fu, X. Yan, Y. Chen, L. Xu, D. Sun et al., Boosting bifunctional oxygen electrocatalysis with 3d graphene aerogel-supported Ni/MnO particles. Adv. Mater. **30**(5), 1704609 (2018). 10.1002/adma.201704609 10.1002/adma.201704609 29235164

[34] D. Xu, N. Wu, K. Le, F. Wang, Z. Wang et al., Bimetal oxide-derived flower-like heterogeneous Co/MnO@C composites with synergistic magnetic–dielectric attenuation for electromagnetic wave absorption. J. Mater. Chem. C **8**(7), 2451–2459 (2020). 10.1039/c9tc05852d

[35] Z. Yang, Y. Qi, F. Wang, Z. Han, Y. Jiang et al., State-of-the-art advancements in photo-assisted CO_2_ hydrogenation: recent progress in catalyst development and reaction mechanisms. J. Mater. Chem. A **8**(47), 24868–24894 (2020). 10.1039/d0ta08781e

[36] Y. Zhang, L. Lu, S. Zhang, Z. Lv, D. Yang et al., Biomass chitosan derived cobalt/nitrogen doped carbon nanotubes for the electrocatalytic oxygen reduction reaction. J. Mater. Chem. A **6**(14), 5740–5745 (2018). 10.1039/c7ta11258k

[37] Y. Li, L. Gai, G. Song, Q. An, Z. Xiao et al., Enhanced properties of CoS_2_/Cu_2_S embedded N/S co-doped mesh-like carbonaceous composites for electromagnetic wave absorption. Carbon **186**, 238 (2022). 10.1016/j.carbon.2021.10.024

[38] Y. Wang, R. Cheng, W.-G. Cui, Z. Lu, Y. Yang et al., Heterostructure design of 3d hydrangea-like Fe_3_O_4_/Fe_7_S_8_@C core-shell composite as a high-efficiency microwave absorber. Carbon **210**, 118043 (2023). 10.1016/j.carbon.2023.118043

[39] W. Wang, K. Nan, H. Zheng, Q. Li, Y. Wang, Ion-exchange reaction construction of carbon nanotube-modified CoNi@MoO_2_/C composite for ultra-intense and broad electromagnetic wave absorption. Carbon **210**, 118074 (2023). 10.1016/j.carbon.2023.118074

[40] P. Hao, Z. Zhao, Y. Leng, J. Tian, Y. Sang et al., Graphene-based nitrogen self-doped hierarchical porous carbon aerogels derived from chitosan for high performance supercapacitors. Nano Energy **15**, 9–23 (2015). 10.1016/j.nanoen.2015.02.035

[41] W. Gu, J. Sheng, Q. Huang, G. Wang, J. Chen et al., Environmentally friendly and multifunctional shaddock peel-based carbon aerogel for thermal-insulation and microwave absorption. Nano-Micro Lett. **13**(1), 102 (2021). 10.1007/s40820-021-00635-1 10.1007/s40820-021-00635-1 PMC8021664 34138342

[42] M. Zhang, H. Ling, T. Wang, Y. Jiang, G. Song et al., An equivalent substitute strategy for constructing 3d ordered porous carbon foams and their electromagnetic attenuation mechanism. Nano-Micro Lett. **14**(1), 157 (2022). 10.1007/s40820-022-00900-x 10.1007/s40820-022-00900-x PMC9346049 35916976

[43] H. Wang, Q. An, Z. Xiao, Y. Tong, L. Guo et al., Marine polysaccharide-based electromagnetic absorbing/shielding materials: design principles, structure, and properties. J. Mater. Chem. A **10**(33), 17023–17052 (2022). 10.1039/d2ta03529d

[44] A. Wang, J. Ni, W. Wang, D. Liu, Q. Zhu et al., MOF Derived Co-Fe nitrogen doped graphite carbon@crosslinked magnetic chitosan micro-nanoreactor for environmental applications: synergy enhancement effect of adsorption−PMS activation. Appl. Catal. B **319**, 121926 (2022). 10.1016/j.apcatb.2022.121926

[45] H. Zhao, Y. Cheng, W. Liu, L. Yang, B. Zhang et al., Biomass-derived porous carbon-based nanostructures for microwave absorption. Nano-Micro Lett. **11**(1), 24 (2019). 10.1007/s40820-019-0255-3 10.1007/s40820-019-0255-3 PMC7770762 34137956

[46] L. Gai, G. Song, Y. Li, W. Niu, L. Qin et al., Versatile bimetal sulfides nanoparticles-embedded N-doped hierarchical carbonaceous aerogels (N-Ni_x_S_y_/Co_x_S_y_@C) for excellent supercapacitors and microwave absorption. Carbon **179**, 111–124 (2021). 10.1016/j.carbon.2021.04.029

[47] L. Gai, Y. Zhao, G. Song, Q. An, Z. Xiao et al., Construction of core-shell PPy@MoS_2_ with nanotube-like heterostructures for electromagnetic wave absorption: assembly and enhanced mechanism. Compos. Part A Appl. Sci. Manuf. **136**, 105965 (2020). 10.1016/j.compositesa.2020.105965

[48] Z. Cai, Y. Ma, K. Zhao, M. Yun, X. Wang et al., Ti_3_C_2_T_x_ MXene/graphene oxide/Co_3_O_4_ nanorods aerogels with tunable and broadband electromagnetic wave absorption. Chem. Eng. J. **462**, 142042 (2023). 10.1016/j.cej.2023.142042

[49] X. Zhang, X. Tian, J. Qiao, X. Fang, K. Liu et al., In-situ fabrication of sustainable-n-doped-carbon-nanotube-encapsulated CoNi heterogenous nanocomposites for high-efficiency electromagnetic wave absorption. Small (2023). 10.1002/smll.202302686 10.1002/smll.202302686 37208798

[50] S. Wu, J. Qiao, Y. Tang, X. Zhang, X. Meng et al., Heterogeneous Cu_9_S_5_/C nanocomposite fibers with adjustable electromagnetic parameters for efficient electromagnetic absorption. J. Colloid Interface Sci. **630**(Pt B), 47–56 (2023). 10.1016/j.jcis.2022.10.075 36327738 10.1016/j.jcis.2022.10.075

[51] Z. Zhang, J. Wang, J. Shang, Y. Xu, Y.J. Wan et al., A through-thickness arrayed carbon fibers elastomer with horizontal segregated magnetic network for highly efficient thermal management and electromagnetic wave absorption. Small **19**(4), e2205716 (2023). 10.1002/smll.202205716 36437045 10.1002/smll.202205716

[52] W.-L. Song, L.-Z. Fan, Z.-L. Hou, K.-L. Zhang, Y. Ma et al., A wearable microwave absorption cloth. J. Mater. Chem. C **5**(9), 2432–2441 (2017). 10.1039/c6tc05577j

[53] X. Meng, S. Dong, Design and construction of lightweight C/Co heterojunction nanofibres for enhanced microwave absorption performance. J. Alloys Compd. **810**, 151806 (2019). 10.1016/j.jallcom.2019.151806

[54] B. Zhao, Z. Bai, H. Lv, Z. Yan, Y. Du et al., Self-healing liquid metal magnetic hydrogels for smart feedback sensors and high-performance electromagnetic shielding. Nano-Micro Lett. **15**(1), 79 (2023). 10.1007/s40820-023-01043-3 10.1007/s40820-023-01043-3 PMC10066054 37002442

[55] G. Chen, H. Liang, J. Yun, L. Zhang, H. Wu et al., Ultrasonic field induces better crystallinity and abundant defects at grain boundaries to develop cus electromagnetic wave absorber. Adv. Mater. (2023). 10.1002/adma.202305586 10.1002/adma.202305586 37565983

[56] H. Liang, L. Zhang, H. Wu, Exploration of twin-modified grain boundary engineering in metallic copper predominated electromagnetic wave absorber. Small **18**(38), e2203620 (2022). 10.1002/smll.202203620 35989098 10.1002/smll.202203620

[57] R. Cui, Y. Li, Y. Huang, W. Wang, C. Wan, Dielectric matching by the unique dynamic dipoles in hybrid organic/inorganic superlattices toward ultrathin microwave absorber. Small (2023). 10.1002/smll.202303008 10.1002/smll.202303008 37485638

[58] K. Li, L. Han, J. Zhang, J. Cheng, Metal-organic framework derived multidimensional carbon/multifluorination epoxy nanocomposite with electromagnetic wave absorption, environmentally adaptive, and blue energy harvesting. Small Struct. (2023). 10.1002/sstr.202300210

[59] T. Xue, Y. Yang, D. Yu, Q. Wali, Z. Wang et al., 3D printed integrated gradient-conductive MXene/CNT/polyimide aerogel frames for electromagnetic interference shielding with ultra-low reflection. Nano-Micro Lett. **15**(1), 45 (2023). 10.1007/s40820-023-01017-5 10.1007/s40820-023-01017-5 PMC9908813 36752927

[60] H. Liang, G. Chen, D. Liu, Z. Li, S. Hui et al., Exploring the Ni 3d orbital unpaired electrons induced polarization loss based on Ni single-atoms model absorber. Adv. Funct. Mater. **33**(7), 2212604 (2022). 10.1002/adfm.202212604

[61] X. Liu, W. Ma, Z. Qiu, T. Yang, J. Wang et al., Manipulation of impedance matching toward 3d-printed lightweight and stiff MXene-based aerogels for consecutive multiband tunable electromagnetic wave absorption. ACS Nano **17**(9), 8420–8432 (2023). 10.1021/acsnano.3c00338 37073866 10.1021/acsnano.3c00338

[62] B.X. Li, Z. Luo, W.G. Yang, H. Sun, Y. Ding et al., Adaptive and adjustable MXene/reduced graphene oxide hybrid aerogel composites integrated with phase-change material and thermochromic coating for synchronous visible/infrared camouflages. ACS Nano **17**(7), 6875–6885 (2023). 10.1021/acsnano.3c00573 36996266 10.1021/acsnano.3c00573

