# Supplementary material for: Nitrogen-Doped Magnetic-Dielectric-Carbon Aerogel for High-Efficiency Electromagnetic Wave Absorption
Source: Nanomicro Lett. 2023 Nov 17;16:16. doi: 10.1007/s40820-023-01244-w (PMC10656410; doi:10.1007/s40820-023-01244-w)
Supplement: Supplementary file 1 — Supplementary file1 (PDF 1691 kb) [file 40820_2023_1244_MOESM1_ESM.pdf]

Supporting Information for

Nitrogen-Doped Magnetic-Dielectric-Carbon Aerogel for High-Efficiency Electromagnetic Wave Absorption

Shijie Wang[1], Xue Zhang[1], Shuyan Hao[1], Jing Qiao[1, 2, *], Zhou Wang[1], Lili Wu[1], Jiurong Liu[1, *], Fenglong Wang[1, 3, *]

[1] Key Laboratory for Liquid-Solid Structural Evolution and Processing of Materials Ministry of Education, Shandong University, Jinan 250061, P. R. China

[2] School of Mechanical Engineering, Shandong University, Jinan 250061, P. R. China

[3] Shenzhen Research Institute of Shandong University, Shenzhen, Guangdong 518057, P. R. China

*Corresponding authors. E-mails: jingqiao@sdu.edu.cn (J.Q); jrliu@sdu.edu.cn (J.R.L); fenglong.wang@sdu.edu.cn (F.L.W)

Supplementary Figures and Tables

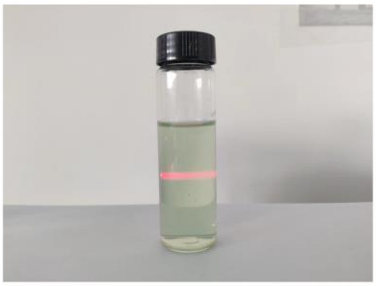

Fig. S1 The Tyndall effect of Ni/Mn-Cs colloidal dispersion

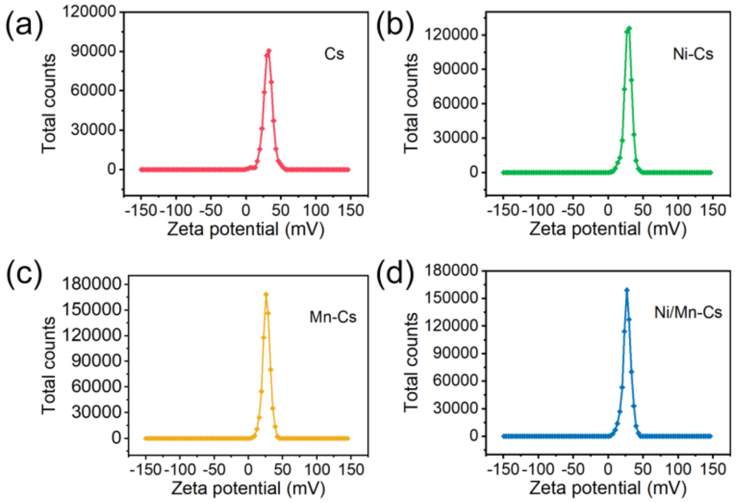

Fig. S2 Zeta potential

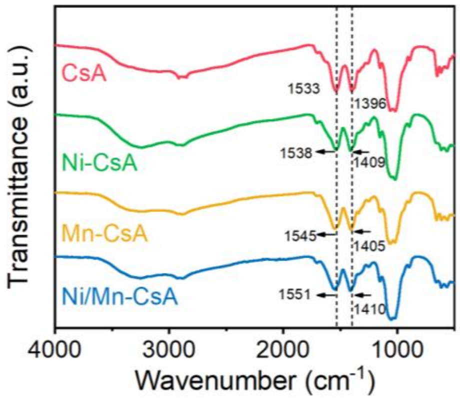

Fig. S3 FT-IR of xerogel before and after adding metal ions

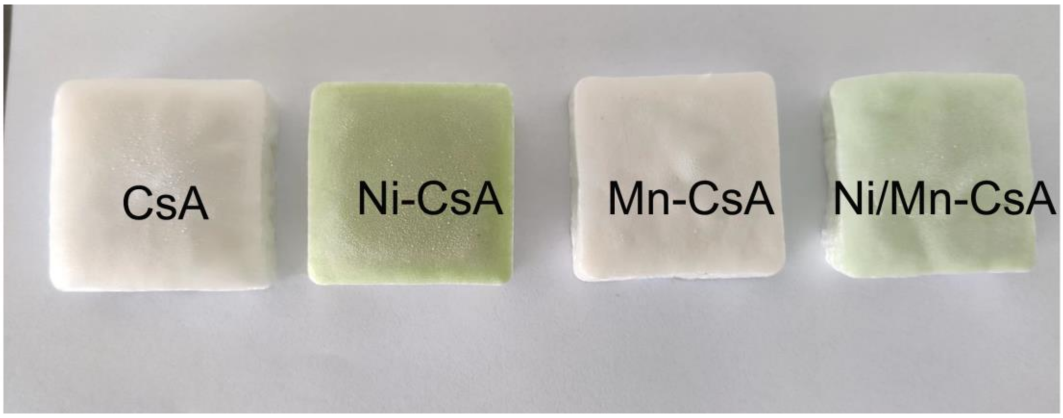

Fig. S4 Chitosan-derived xerogel before and after adding metal ions

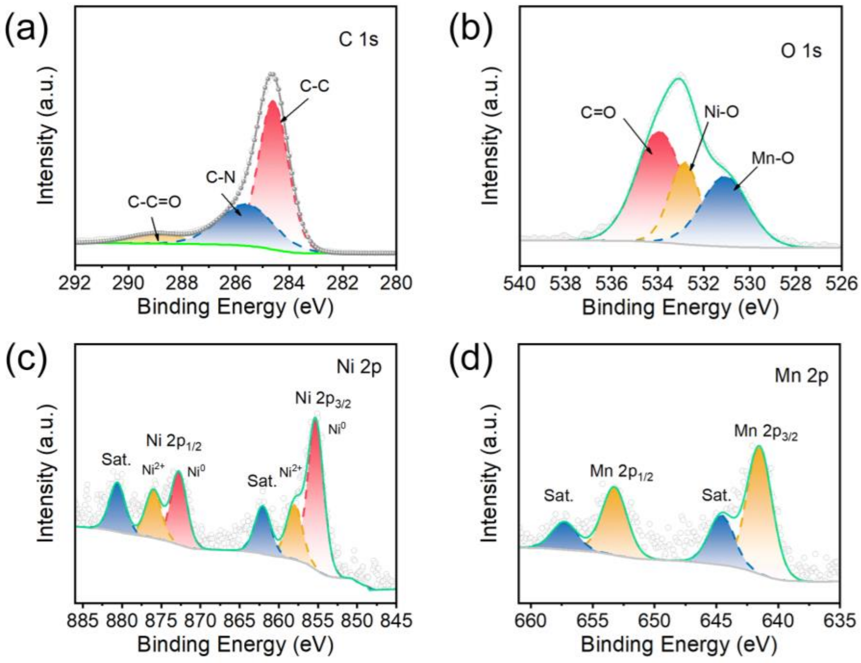

Fig. S5 XPS spectra of Ni/MnO-CA, **a** C 1s. **b** O 1s. **c** Ni 2p and **d** Mn 2p

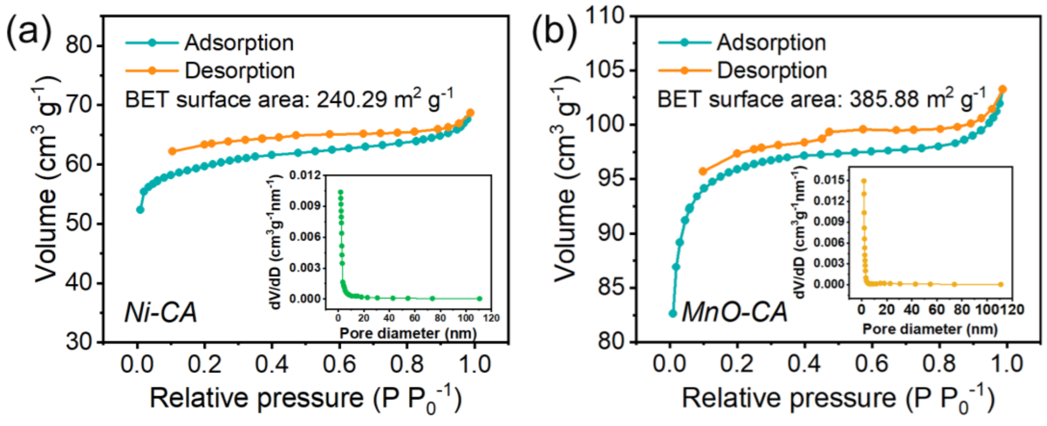

Fig. S6 BET surface area and pore size distribution. **a** Ni-CA and **b** MnO-CA

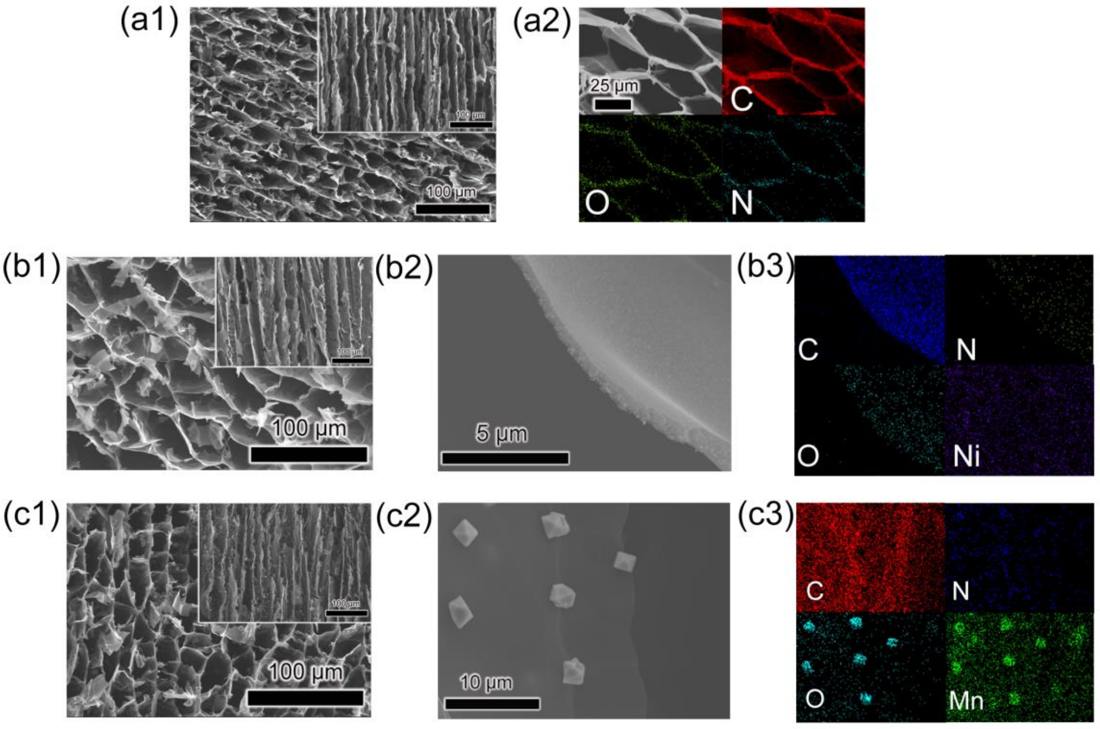

Fig. S7 The field emission scanning electron microscope (FE-SEM). **a1** CA. **b1–2** Ni-CA and **c1–2** MnO-CA, the inset was parallel to the direction of ice crystal. EDS mappings, **a2** CA. **b3** Ni-CA and **c3** MnO-CA

According to the weight loss in TGA curves, the carbon contents in aerogels could be calculated by the following equations, where M was the relative molecular mass of the corresponding substance.

$$wt\%_{\text{Carbon}} = 1 - wt\%_{\text{Residue}} \times \frac{M_{\text{Ni}}}{M_{\text{NiO}}} \quad \text{Eq. S1}$$

$$wt\%_{\text{Carbon}} = 1 - wt\%_{\text{Residue}} \times \frac{3M_{\text{MnO}}}{M_{\text{Mn}_3\text{O}_4}} \qquad \text{Eq. S2}$$

$$wt\%_{\text{Carbon}} = 1 - wt\%_{\text{Residue}} \times \frac{M_{\text{Ni}} + 3M_{\text{MnO}}}{M_{\text{NiO}} + M_{\text{Mn}_3\text{O}_4}} \qquad \text{Eq. S3}$$

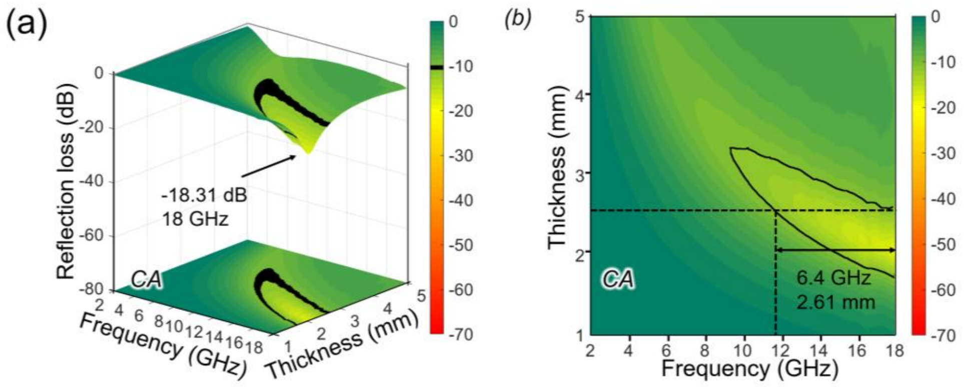

Fig. S8 The 3D RL plots. **a** CA. The 2D contours of RL values versus frequency and thickness. **b** CA

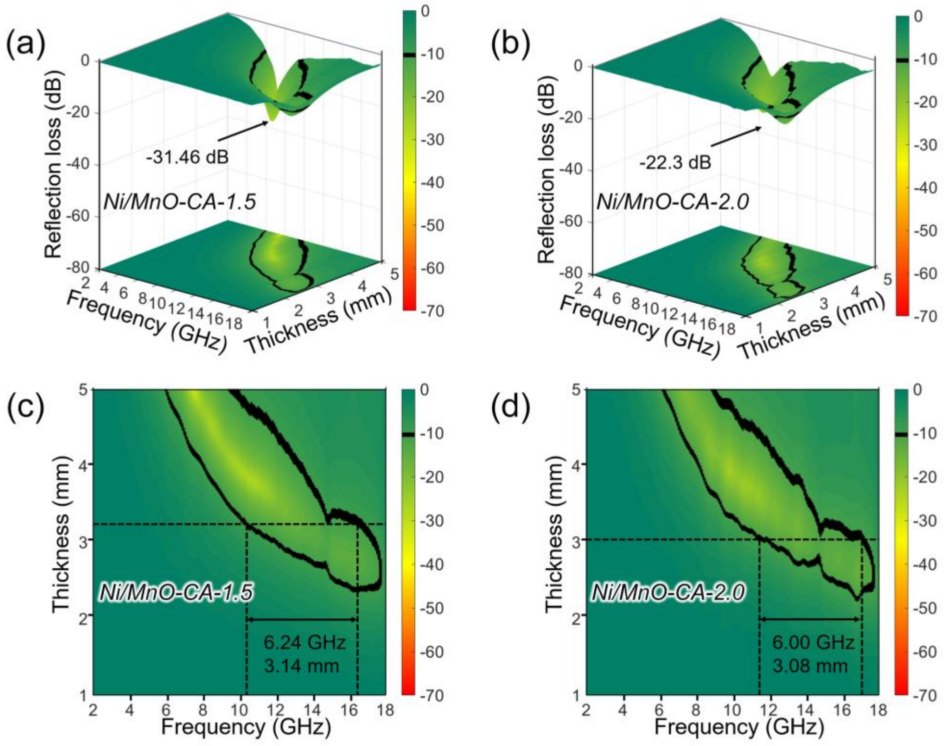

Fig. S9 The 3D RL plots. **a** Ni/MnO-CA-1.5 and **b** Ni/MnO-CA-2.0. The 2D contours of RL values versus frequency and thickness. **c** Ni/MnO-CA-1.5 and **d** Ni/MnO-CA-2.0

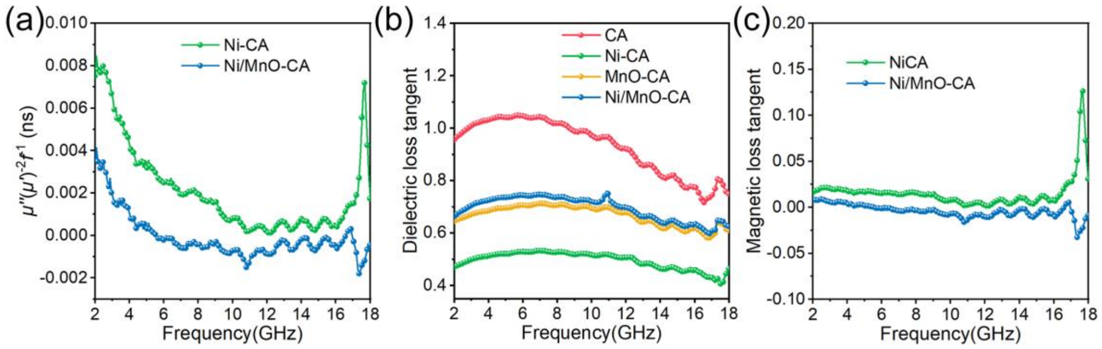

Fig. S10 a C_0 values. **b** Dielectric loss tangent and **c** Magnetic loss tangent

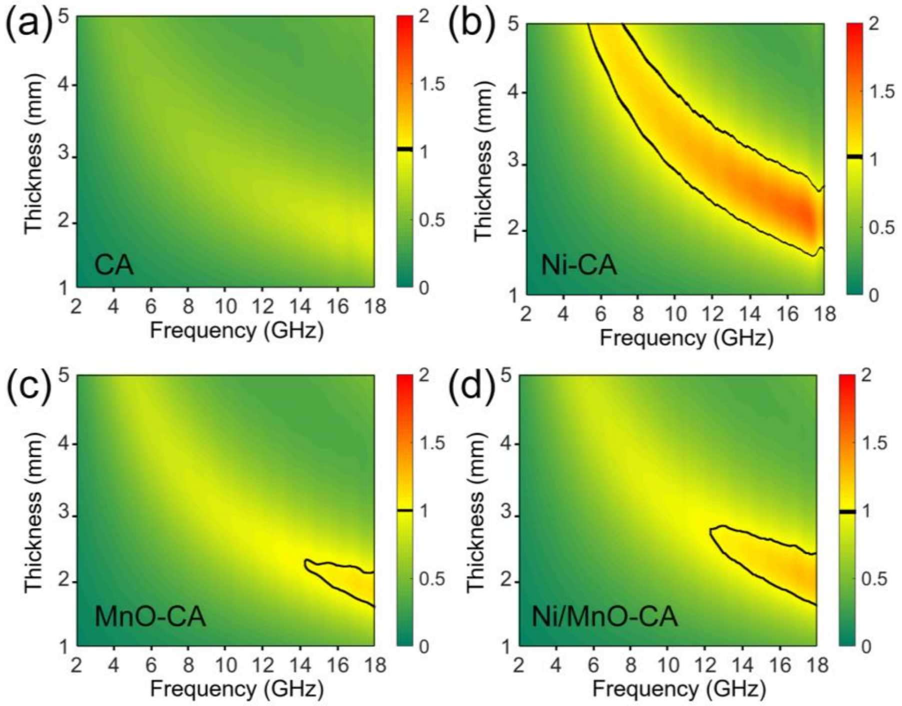

Fig. S11 2D impedance matching plots

Electromagnetic Formulas

According to transmission line theory, the reflection loss (RL) in this work is calculated by the followed formulars:

$$Z_{in} = Z_0 \sqrt{\frac{\mu_r}{\varepsilon_r}} \tanh(j \frac{2\pi fd}{c} \sqrt{\mu_r \varepsilon_r}) \qquad \text{Eq. S4}$$

$$RL = 20 \log \left| \frac{Z_{in} - Z_0}{Z_{in} + Z_0} \right| \qquad \text{Eq. S5}$$

where Z_{in} and Z_0 represent the impendence of the absorbing coating and air. In addition,

μ_r, ε_r, f, d and c refer to complex permeability, complex permittivity, frequency, thickness and velocity of light, respectively.

In generally, the polarization-relaxation process of electromagnetic wave absorption is evaluated by the Cole–Cole plot. The plot based on modified Debye theory is introduced by the following equations:

$$\varepsilon' = \varepsilon_\infty + \frac{\varepsilon_s - \varepsilon_\infty}{1+(2\pi f)^2\tau^2} \quad \text{Eq. S6}$$

$$\varepsilon'' = \frac{2\pi f\tau(\varepsilon_s - \varepsilon_\infty)}{1+(2\pi f)^2\tau^2} + \frac{\sigma}{2\pi f\varepsilon_0} \quad \text{Eq. S7}$$

where ε_s, ε_∞ and ε_0 represent permittivity at electrostatic field, permittivity at high frequency limit and permittivity of vacuum, respectively; τ, f and σ are the polarization relaxation time, frequency and conductivity, respectively. The above two equations can be combined and simplified as below:

$$(\varepsilon' - \frac{\varepsilon_s + \varepsilon_\infty}{2})^2 + (\varepsilon'' - \frac{\sigma}{2\pi f\varepsilon_0})^2 = (\frac{\varepsilon_s - \varepsilon_\infty}{2})^2 \quad \text{Eq. S8}$$

In the Cole–Cole plot, the semicircle indicates the presence of polarization relaxation, while the tail-like straight line represents the conductivity.

The loss capacity is calculated by the attenuation coefficient (α):

$$\alpha = \frac{\sqrt{2}\pi f}{c}\sqrt{(\mu''\varepsilon'' - \mu'\varepsilon') + \sqrt{(\mu''\varepsilon'' - \mu'\varepsilon')^2 + (\mu'\varepsilon'' + \mu''\varepsilon')^2}} \quad \text{Eq. S9}$$

Radar cross section (RCS) simulation by *CST microwave studio*:

RCS is used to simulate the far-field response of absorber to illustrate the actual stealth performance of the material. Herein, the model construction and excitation configuration are as follows: The model width of the perfect electric conductor (PEC) plate is 200.0 mm × 200.0 mm, and the thickness is 2.0 mm. The thickness of coating (the aerogel-paraffin layer, as a absorber) is 3.0 mm. The far field, the incident electromagnetic wave and the position of the model are shown below (Fig. S12). What needs to be explained in the figure is that the incident electromagnetic wave is vertically polarized wave. For the setting of polarized wave, the incident azimuth angles are restricted within the condition of "–60° ≤ phi ≤ 60°, theta = 90°". In addition, we chose the 6 GHz (C band), 9 GHz (X band) and 15 GHz (Ku band) as the frequency of the far-field monitor. The RCS values are described by the formular:

$$\sigma(\text{dB m}^2) = 10\log(\frac{4\pi S}{\lambda^2}\left|\frac{E_s}{E_i}\right|)^2 \quad \text{Eq. S10}$$

where S is the area of the simulated plate and λ is the wavelength of incident wave; E_i and E_s represent the electric field strength of the incident and scattered waves, respectively.

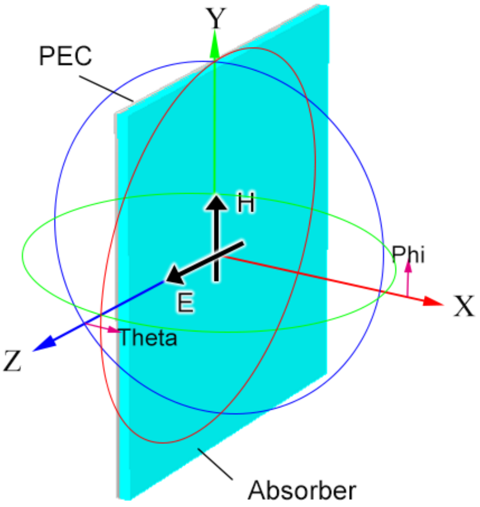

Fig. S12 Illustration of the CST simulation model

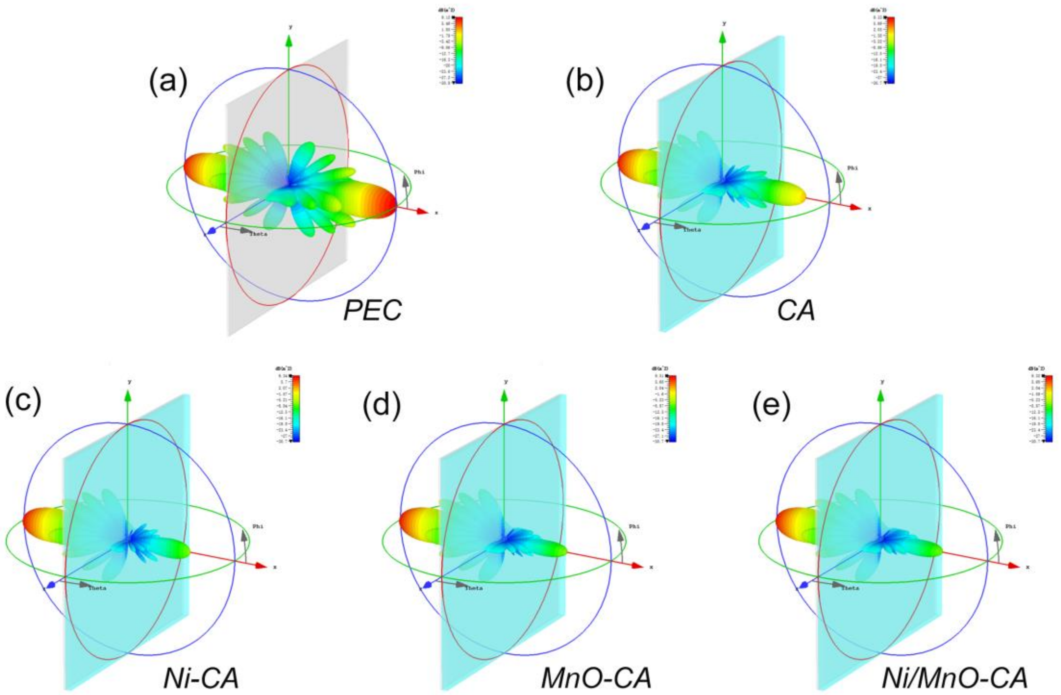

Fig. S13 The 3D far-field response of RCS simulations at 5 GHz of **a** PEC, **b** CA, **c** Ni-CA, **d** MnO-CA and **e** Ni/MnO-CA

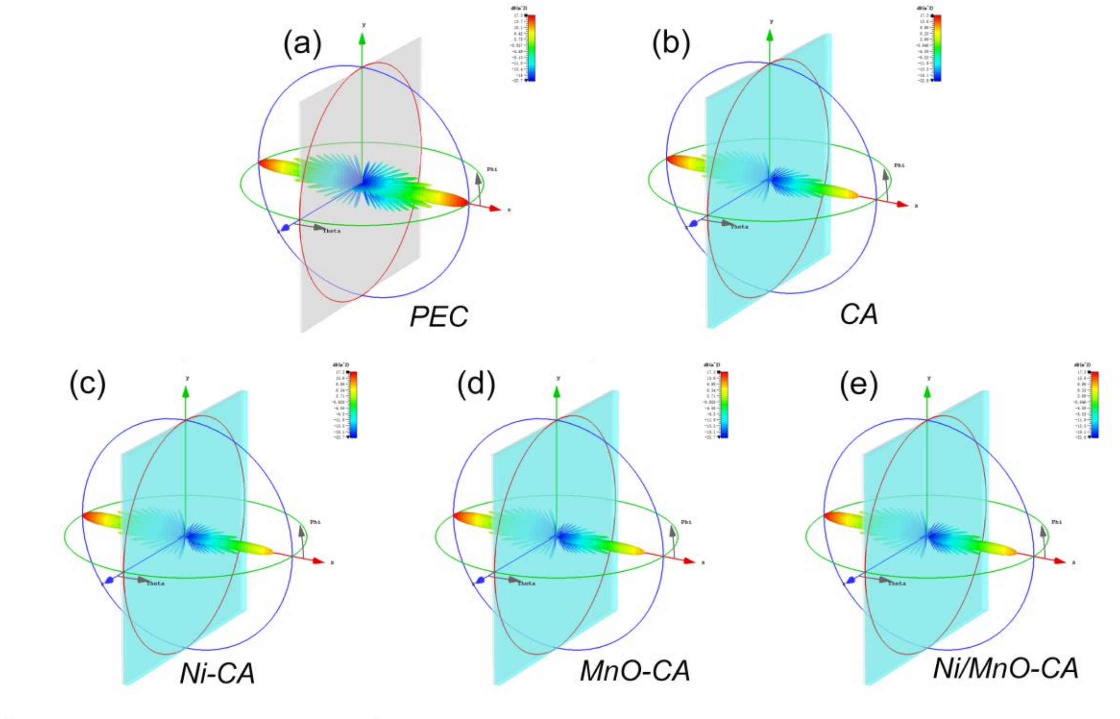

Fig. S14 The 3D far-field response of RCS simulations at 15 GHz of **a** PEC, **b** CA, **c** Ni-CA, **d** MnO-CA and **e** Ni/MnO-CA

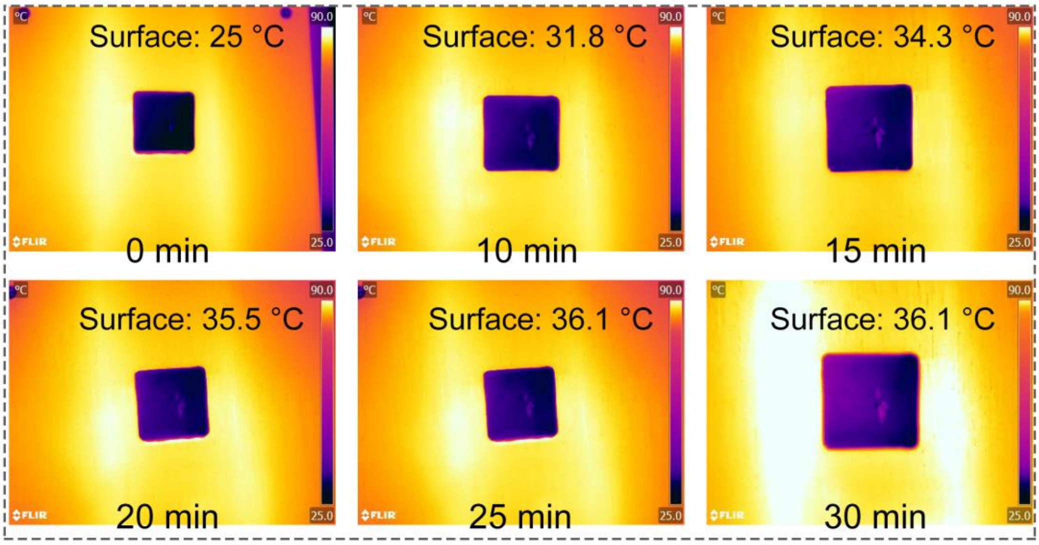

Fig. S15 Infrared thermal images of CA on a constant temperature heating plate of 90 °C

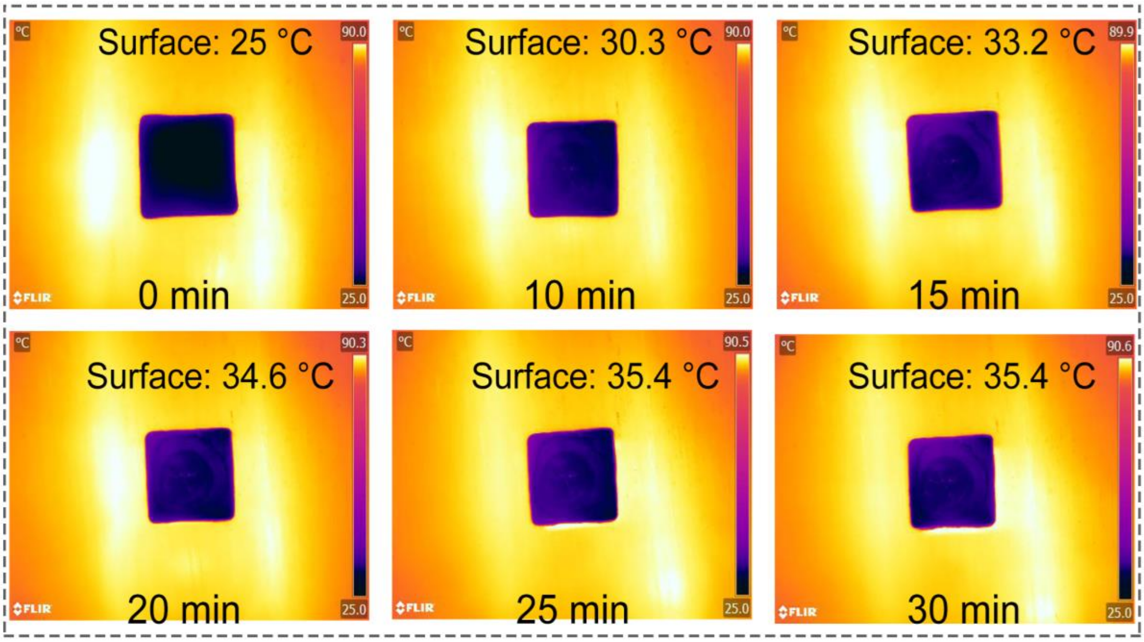

Fig. S16 Infrared thermal images of Ni-CA on a constant temperature heating plate of 90 °C

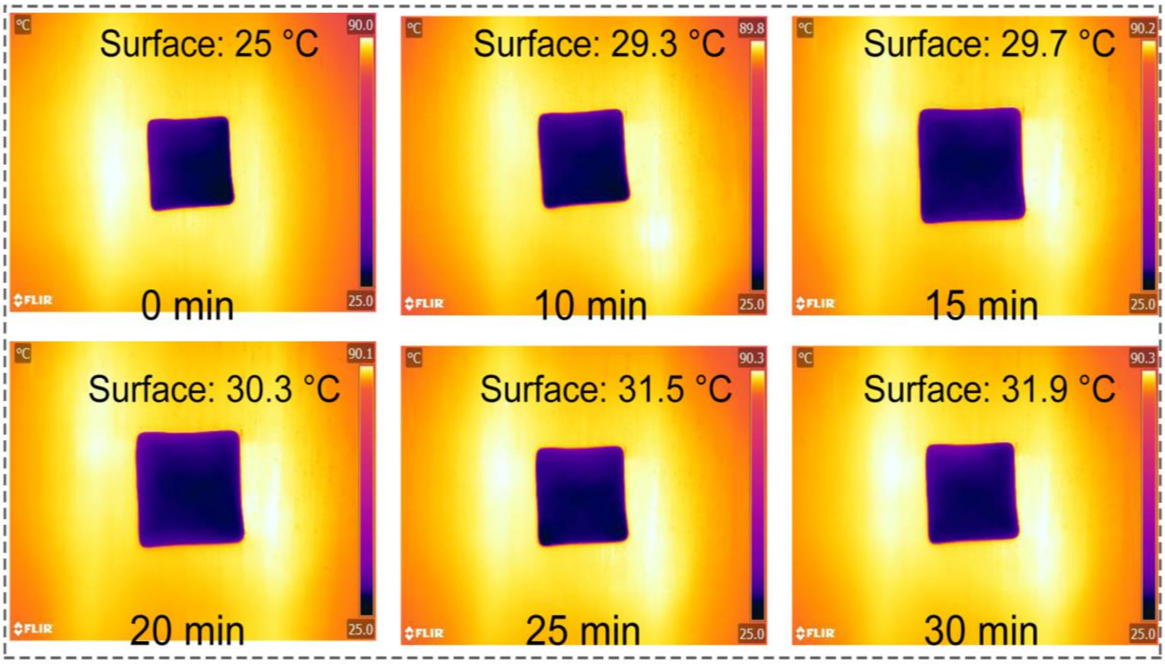

Fig. S17 Infrared thermal images of MnO-CA on a constant temperature heating plate of 90 °C

Table S1 Detailed information for the performance comparison with other absorbers

Materials	EAB (GHz)	RL_{min} (dB)	Thickness (mm)	Filler Rate (wt%)	Refs.
MnO/Co/C	5.30	–68.89	2.64	50	[S1]
Ni/TiO_2/C	6.70	–74.50	2.00	15	[S2]
Cu-NC-10	5.25	–63.80	2.01	35	[S3]
MoO_2/CoNi/NPC	4.72	–54.00	1.97	35	[S4]
NCFs@WS_2	6.24	–81.10	3.5	10	[S5]
Co/MnO/CNTs	5.36	–58.00	2.65	35	[S6]
MXene/graphene oxide/Co_3O_4	6.88	–71.87	2.07	17	[S7]
NiCo/C/CNT/rGO	7.6	–58.80	1.80	20	[S8]
NRGO/MWVNTs	5.20	–53.30	3.46	15	[S9]
NiO/$NiFe_2O_4$@N-rGA	6.58	–57.70	2.13	15	[S10]
Cu/CuO/C	5.5	–44.00	2.40	50	[S11]
ZnCo2O4@ZIF-67	5.79	–59.18	1.97	30	[S12]
Fe_3O_4-SnO_2	5.60	–66.50	3.00	30	[S13]
**Ni/MnO-CA**	**7.36**	**–64.09**	**2.53**	**10**	**This work**

Supplementary References

[S1] X. Zhang, X. Tian, C. Liu, J. Qiao, W. Liu et al., MnCo-MOF-74 derived porous MnO/Co/C heterogeneous nanocomposites for high-efficiency electromagnetic wave absorption. Carbon **194**, 257–266 (2022). https://doi.org/10.1016/j.carbon.2022.04.001

[S2] J. Qiao, X. Zhang, C. Liu, L. Lyu, Z. Wang et al., Facile fabrication of Ni embedded TiO_2/C core-shell ternary nanofibers with multicomponent functional synergy for efficient electromagnetic wave absorption. Compos. Part B Eng. **200**, 108343 (2020). https://doi.org/10.1016/j.compositesb.2020.108343

[S3] H. Liang, L. Zhang, H. Wu, Exploration of twin-modified grain boundary engineering in metallic copper predominated electromagnetic wave absorber. Small **18**, e2203620 (2022). https://doi.org/10.1002/smll.202203620

[S4] N. Zhang, K. Zhang, W. Chen, Y. Wang, T. Liu et al., Constructing hierarchical hollow flower-like MoO_2/CoNi/NPC microspheres via water-in-oil system assisted supramolecular self-assembly for high-efficient electromagnetic wave absorption. J. Alloys Compd. 170684 (2023). https://doi.org/10.1016/j.jallcom.2023.170684

[S5] X. Liu, S. Zhang, M. Yu, X. Zhao, Y. Jia et al., WS_2 nanosheets anchored on N-doped carbon fibers for superior electromagnetic wave absorption. Chem. Eng. J. **465**, 142932 (2023). https://doi.org/10.1016/j.cej.2023.142932

[S6] W. Xue, G. Yang, S. Bi, J. Zhang, Z.-L. Hou, Construction of caterpillar-like hierarchically structured Co/MnO/CNTs derived from MnO_2/ZIF-8@ZIF-67 for electromagnetic wave absorption. Carbon **173**, 521–527 (2021). https://doi.org/10.1016/j.carbon.2020.11.016

[S7] Z. Cai, Y. Ma, K. Zhao, M. Yun, X. Wang et al., $Ti_3C_2T_x$ MXene/graphene oxide/Co_3O_4 nanorods aerogels with tunable and broadband electromagnetic wave absorption. Chem. Eng. J. **462**, 142042 (2023). https://doi.org/10.1016/j.cej.2023.142042

[S8] L. Yang, Y. Wang, Z. Lu, R. Cheng, N. Wang et al., Construction of multi-dimensional NiCo/C/CNT/rGO aerogel by MOF derivative for efficient microwave absorption. Carbon **205**, 411–421 (2023). https://doi.org/10.1016/j.carbon.2023.01.057

[S9] R. Shu, Z. Wan, J. Zhang, Y. Wu, J. Shi, Synergistically assembled nitrogen-doped reduced graphene oxide/multi-walled carbon nanotubes composite aerogels with superior electromagnetic wave absorption performance. Compos. Sci. Technol. **210**, 108818 (2021). https://doi.org/10.1016/j.compscitech.2021.108818

[S10] Q. Wang, J. Wang, Y. Zhao, Y. Zhao, J. Yan et al., NiO/$NiFe_2O_4$@N-doped reduced graphene oxide aerogel towards the wideband electromagnetic wave absorption: Experimental and theoretical study. Chem. Eng. J. **430**, 132814 (2022). https://doi.org/10.1016/j.cej.2021.132814

[S11] Y. Guo, Q. Chang, Z. Shi, J. Xie, J. Yun et al., Regulating conduction and polarization losses by adjusting bonded N in N-doped Cu/CuO/C composites. J. Colloid Interface Sci. **639**, 444–453 (2023). https://doi.org/10.1016/j.jcis.2023.02.093

[S12] Y. Zhao, W. Wang, Q. Wang, H. Zhao, P. Li et al., Construction of excellent electromagnetic wave absorber from multi-heterostructure materials derived from $ZnCo_2O_4$ and ZIF-67 composite. Carbon **185**, 514–525 (2021). https://doi.org/10.1016/j.carbon.2021.09.049

[S13] R. Kuchi, V. Dongquoc, P.C. Van, D. Kim, J.-R. Jeong, Synthesis of porous Fe_3O_4-SnO_2 core-void-shell nanocomposites as high-performance microwave absorbers. J. Environ. Chem. Eng. **9**, 106585 (2021). https://doi.org/10.1016/j.jece.2021.106585
